# Spatiotemporal exposure modeling of ambient erythemal ultraviolet radiation

**DOI:** 10.1186/s12940-016-0197-x

**Published:** 2016-11-24

**Authors:** Trang VoPham, Jaime E. Hart, Kimberly A. Bertrand, Zhibin Sun, Rulla M. Tamimi, Francine Laden

**Affiliations:** 1 Department of Epidemiology, Harvard T.H. Chan School of Public Health, Boston, MA USA; 2 Channing Division of Network Medicine, Department of Medicine, Brigham and Women’s Hospital and Harvard Medical School, Boston, MA USA; 3 Exposure, Epidemiology, and Risk Program, Department of Environmental Health, Harvard T.H. Chan School of Public Health, Boston, MA USA; 4 Slone Epidemiology Center at Boston University, Boston, MA USA; 5 U.S. Department of Agriculture UV-B Monitoring and Research Program, Colorado State University, Fort Collins, CO USA

**Keywords:** Ultraviolet radiation, Erythemal ultraviolet radiation, Kriging, Geostatistics, Exposure model, Area-to-point residual kriging

## Abstract

**Background:**

Ultraviolet B (UV-B) radiation plays a multifaceted role in human health, inducing DNA damage and representing the primary source of vitamin D for most humans; however, current U.S. UV exposure models are limited in spatial, temporal, and/or spectral resolution. Area-to-point (ATP) residual kriging is a geostatistical method that can be used to create a spatiotemporal exposure model by downscaling from an area- to point-level spatial resolution using fine-scale ancillary data.

**Methods:**

A stratified ATP residual kriging approach was used to predict average July noon-time erythemal UV (UV_Ery_) (mW/m^2^) biennially from 1998 to 2012 by downscaling National Aeronautics and Space Administration (NASA) Total Ozone Mapping Spectrometer (TOMS) and Ozone Monitoring Instrument (OMI) gridded remote sensing images to a 1 km spatial resolution. Ancillary data were incorporated in random intercept linear mixed-effects regression models. Modeling was performed separately within nine U.S. regions to satisfy stationarity and account for locally varying associations between UV_Ery_ and predictors. Cross-validation was used to compare ATP residual kriging models and NASA grids to UV-B Monitoring and Research Program (UVMRP) measurements (gold standard).

**Results:**

Predictors included in the final regional models included surface albedo, aerosol optical depth (AOD), cloud cover, dew point, elevation, latitude, ozone, surface incoming shortwave flux, sulfur dioxide (SO_2_), year, and interactions between year and surface albedo, AOD, cloud cover, dew point, elevation, latitude, and SO_2_. ATP residual kriging models more accurately estimated UV_Ery_ at UVMRP monitoring stations on average compared to NASA grids across the contiguous U.S. (average mean absolute error [MAE] for ATP, NASA: 15.8, 20.3; average root mean square error [RMSE]: 21.3, 25.5). ATP residual kriging was associated with positive percent relative improvements in MAE (0.6–31.5%) and RMSE (3.6–29.4%) across all regions compared to NASA grids.

**Conclusions:**

ATP residual kriging incorporating fine-scale spatial predictors can provide more accurate, high-resolution UV_Ery_ estimates compared to using NASA grids and can be used in epidemiologic studies examining the health effects of ambient UV.

**Electronic supplementary material:**

The online version of this article (doi:10.1186/s12940-016-0197-x) contains supplementary material, which is available to authorized users.

## Background

Ultraviolet (UV) radiation is a ubiquitous environmental exposure classified as a Group 1 human carcinogen according to the International Agency for Research on Cancer (IARC) [1]. UV radiation is emitted by the sun and comprised of UV-A (315–400 nm), UV-B (280–315 nm), and UV-C (100–280 nm) wavelengths [2]. The majority of UV-A reaches the Earth’s surface, while 90% of UV-B and virtually all UV-C is absorbed by ozone and other atmospheric constituents [2]. Epidemiologic studies have linked high levels of UV-A and UV-B exposure with an increased risk of developing skin cancer [3]. On the other hand, the primary source of vitamin D for most humans is UV-B [4]. UV-B penetrates the skin, which converts 7-dehydrocholesterol to previtamin D_3_ and subsequently vitamin D_3_ [5]. Vitamin D deficiency (serum 25-hydroxyvitamin D (25(OH)D) levels <20 nm/mL) affects approximately 50% of the world’s population [6], and has been associated with increased risks of osteoporosis, cardiovascular disease, and cancers [5, 7].

Given the multifaceted roles UV, and in particular UV-B, may play in both adversely affecting and promoting human health, accurate exposure assessment is critical to further elucidating its exact impact on health outcomes in population-based studies. The amount of UV reaching the Earth’s surface is affected by many factors, including ozone, aerosol optical depth (AOD), altitude (elevation is also used to define relative height), and cloud cover [2, 8]. Several UV exposure models have been developed for use in studies examining the association between UV and human health outcomes [9–11]. However, these models are limited in their spatial, temporal, and/or spectral resolution, likely contributing to substantial exposure measurement error. Scotto et al. [10] compiled a U.S. state-level composite measure of UV-B radiation averaged from 1974 to 1987, created using altitude, cloud cover, and latitude. Tatalovich et al. [11] developed a 1 km resolution UV exposure model using thin-plate smoothing splines to interpolate average daily total global solar radiation (also referred to as global horizontal irradiance [GHI] [12]), incorporating 1 km elevation and the location of National Solar Radiation Database stations. Although GHI represents all shortwave radiation (including near-ultraviolet to near-infrared wavelengths, 300–2500 nm [13]), and is expected to be correlated with UV-B, this metric lacks specificity if UV-B is the exposure of interest. Previous epidemiologic studies have also used U.S. National Aeronautics and Space Administration (NASA) erythemal UV (UV_Ery_) estimated from the Total Ozone Mapping Spectrometer (TOMS) satellite sensor [14–22]. TOMS UV_Ery_ remote sensing images are available at a daily temporal resolution, estimated using UV irradiance reaching the Earth’s surface that is deduced from measured UV irradiance entering the atmosphere and TOMS total ozone and surface reflectivity information, and weighted by a model of susceptibility of Caucasian skin to sunburn (i.e., erythema) [23]. However, this dataset is limited due to its coarse spatial resolution of approximately 1° latitude × 1.25° longitude.

Area-to-point (ATP) residual kriging (also referred to as ATP regression kriging) is a geostatistical interpolation method capable of downscaling spatial data, or transitioning from a coarse area source unit to a relatively finer point-level spatial resolution [24, 25]. In practice, ATP residual kriging has been conducted when a regression-based prediction model is associated with a low coefficient of determination (R^2^), suggesting that the predictors alone do not explain an adequate amount of the variance in the outcome. Furthermore, if a spatial structure or correlation is observed in the residuals of the regression model (visualized using a variogram), this spatial information can be incorporated into modeling to improve downscaling [26]. ATP residual kriging also satisfies the coherence property, where the attribute value of a variable for a given source area is equal to the average of the downscaled, predicted values of the points discretizing the source area. There have been several applications of this method to downscaling precipitation, legacy soil, population density, Moderate Resolution Imaging Spectroradiometer (MODIS) imagery, and pan-sharpening [26–30]. However, to the best of our knowledge, ATP residual kriging has not been applied to downscale surface UV radiation.

Our goal was to develop an improved nationwide spatiotemporal exposure model of UV_Ery_ from 1998 to 2012 with a higher level of spatial and temporal resolution compared to existing models.

## Methods

### Overview of exposure modeling methodology

ATP residual kriging was used to create a spatiotemporal exposure model of average July noon-time UV_Ery_ (mW/m^2^) biennially from 1998 to 2012. The study area was the contiguous U.S. The source unit or area variable being downscaled was NASA TOMS and OMI UV_Ery_ gridded remote sensing images, referred to as grids from here onward (Table 1). The target scale was 1 km, which determined how many points discretized each grid. Each discretizing point also represents a prediction point, or a location at which UV_Ery_ was predicted. A k-nearest neighborhood around each prediction point was defined (*n* = 16 was used for this study), representing the grids neighboring a prediction point that would be used in later kriging steps. Ancillary data included variables known to be associated with UV gathered from previous literature: surface albedo, aerosol optical depth (AOD), cloud cover, dew point, elevation, ozone, surface incoming shortwave (SIS) flux, sulfur dioxide (SO_2_), and latitude (Table 1) [2, 8, 31–35]. All data were preprocessed for analysis in a geographic information system (GIS) using ArcGIS (Esri, Redlands, CA). UV_Ery_ and ancillary data were both joined with the prediction points and aggregated to the grid level. Segmentation using IDRISI Selva was performed to create nine regions in the contiguous U.S. within which to conduct stratified kriging (Clark Labs, Worcester, MA) [30, 36]. Separately for each region, a grid-level random intercept linear mixed-effects regression model was built (random intercept for grid) using data from 1998 to 2012 (biennially). Grid-level residuals from each model were calculated and a grid-level variogram of the residuals was estimated separately for each year. ATP residual kriging was performed using simple kriging in SpaceStat to downscale the grid-level residuals each year (BioMedware, Ann Arbor, MI). Kriging weights for the residual value of each grid neighboring a given prediction point were calculated using the area-to-area (ATA) covariance and ATP covariance. ATA and ATP covariances require a point support variogram, which was estimated by applying variogram deconvolution to the grid-level variogram [37]. The downscaled residual value at each prediction point was calculated as the sum of the products of the kriging weights and residual values for each neighboring grid. The downscaled UV_Ery_ value at each prediction point was calculated by adding the downscaled residual value to the grid-level regression equation, which is assumed to be representative of the relationship between the predictors and UV_Ery_ at the point level [26]. This process was performed for each prediction point each year, separately for each region.

**Table 1:** Data sources used in ATP residual kriging to downscale NASA UV_Ery_ in the U.S. (1998–2012)

Variable	Unit	Spatial resolution	Temporal resolution	Source
Outcome: UV_Ery_ grids	mW/m^2^	Approx. 100 × 100 km	Daily	NASA TOMS/OMI^a^
Predictors				
Surface albedo	1	70 × 50 km	Monthly	MERRA
Aerosol optical depth (AOD)	1	70 × 50 km	Daily	MERRA
Cloud cover	1	70 × 50 km	Daily	MERRA
Dew point	°F	4 × 4 km	Monthly	PRISM
Elevation	m	10 × 10 m	Updated^b^	USGS 3DEP
Latitude	km	1 × 1 km	n/a	Manually created
Ozone	Dobson	70 × 50 km	Monthly	MERRA
Surface incoming shortwave flux (SIS)	W/m^2^	70 × 50 km	Monthly	MERRA
Sulfur dioxide (SO_2_)	kg/m^2^	70 × 50 km	Monthly	MERRA

Abbreviations: *3DEP* 3D Elevation Program, *MERRA* Modern Era Retrospective Analysis for Research and Applications, *NASA* National Aeronautics and Space Administration, *OMI* Ozone Monitoring Instrument, *PRISM* Parameter elevation Regression on Independent Slopes Model, *TOMS* Total Ozone Mapping Spectrometer, *USGS* U.S. Geological Survey

^a^For the TOMS sensor, the following data products were downloaded corresponding to the Nimbus-7 and Earth Probe satellites: TOMSN7L3:Erythemal_timeAveraged and TOMSEPL3:Erythemal_timeAveraged. The following data product was downloaded from the OMI sensor (Aura satellite): OMUVBd:ErythemalDoseRate_timeAveraged

^b^USGS 3DEP data are continuously updated as new elevation sources are acquired. Data from August 2015 were acquired for this study

### Data sources and preprocessing: UV_Ery_ and ancillary data

UV_Ery_ gridded remote sensing images were acquired from the NASA TOMS and OMI satellite sensors (Table 1). Monthly average July noon-time UV_Ery_ (mW/m^2^) images from the TOMS sensor onboard the Earth Probe satellite from 1998 to 2004 [9] and from the OMI sensor onboard the Aura satellite from 2006 to 2012 [38] were used for modeling. Our study modeled UV_Ery_ in July as previous epidemiologic studies have examined July UV_Ery_ exposure [14–16, 39], and during July, UV_Ery_ is strongest, aerosols and other noise factors are less influential, and satellite-based measures (e.g., TOMS) are in better agreement with ground-based measures [15]. Images from July 1980 and 1990 from the TOMS sensor onboard the Nimbus-7 satellite, in addition to images from 2000 to 2010, were used for segmentation described later, selected due to our anticipated creation of an exposure model beginning in 1980. UV_Ery_ incorporates information regarding both the levels of the different UV wavelengths and their relative effectiveness to induce erythema on Caucasian skin using a model proposed by McKinlay and Diffey, adopted as a standard by the Commission Internationale de l’Eclairage (CIE) [40, 41]. Both UV-A and UV-B radiation are included in the calculation, although shorter UV-B wavelengths are weighted more. The algorithm used to calculate UV_Ery_ from UV irradiance entering the atmosphere, ozone, and reflectivity data from the TOMS and OMI sensors is identical (personal communication, Nickolay Krotkov, NASA, 7/16/15). The original spatial resolution of TOMS and OMI images is 50 × 50 km and 13 × 24 km, respectively [42]. A gridding algorithm is applied to combine TOMS and OMI measurements into a fixed global grid [43], where each grid is approximately 111 km north to south and 75–101 km east to west [16]. TOMS and OMI UV_Ery_ products are available at a daily temporal resolution. The NASA Web Coverage Service (WCS) was used to download time-averaged (average July) UV_Ery_ products each year (personal communication, Wenli Yang, NASA, 6/15/15). The OMI OMUVBd erythemal dose rate product was downloaded for comparability with the TOMS TOMSEPL3 and TOMSN7L3 erythemal products, which do not assume a clear sky and are calculated at local noon (personal communication, James E. Johnson, NASA, 6/17/15). All rasters were reprojected to the contiguous U.S. Albers equal area conic coordinate system (NAD83 datum; USGS version) in ArcGIS.

### Ancillary data

Ancillary data, identified from prior research as being associated with UV and/or used in previous modeling, were included in UV_Ery_ modeling if the data were time-varying (if applicable) and of a finer spatial resolution compared to the NASA UV_Ery_ grids that were downscaled [2, 31–35] (Table 1). The average July value was downloaded for each dataset (if applicable) to match the time period of the NASA TOMS and OMI images in this study. Surface albedo, AOD, cloud cover, ozone, SIS, and SO_2_ were acquired from the NASA Modern-Era Retrospective Analysis for Research and Applications (MERRA), an atmospheric reanalysis of the satellite era using the Goddard Earth Observing System Model, Version 5 (GOES-5) and its Atmospheric Data Assimilation System (ADAS), version 5.2.0 [44]. HDF-EOS files were converted to rasters and reprojected. Surface albedo refers to the reflectivity of the Earth’s surface, or the ratio of reflected irradiance to incident irradiance specifically for horizontal surfaces [2]. AOD is a measure of the aerosols (e.g., haze) distributed within a column of air from the Earth’s surface to the top of the atmosphere [45]. Cloud cover is defined in MERRA as the average proportion of the pixel associated with clouds. Ozone is a gas found in trace amounts in the stratosphere, as well as in the troposphere and at ground level [46]. SIS is the shortwave radiation flux reaching a horizontal unit of the Earth’s surface [47]. SO_2_ is a gas produced from volcanoes and anthropogenic activities (e.g., burning fossil fuels, refineries, metal smelting, and power plants) that can be found near the Earth’s surface and in the free troposphere and stratosphere [48].

Elevation data (seamless 1/3 arc-second [10 m] data for the contiguous U.S.) were acquired from the U.S. Geological Survey (USGS) 3D Elevation Program (3DEP) [49]. All 3DEP grids were mosaicked together and reprojected. Dew point (°C) was acquired from the Parameter elevation Regression on Independent Slopes Model (PRISM) Climate Group [50]. ASCII files were converted to rasters, reprojected, converted to °F, and rescaled to 1 unit equaling 1 °F. A latitude raster file was created using a 1 km fishnet grid, where the latitude value was equivalent to the Y coordinate of the projected coordinate system.

### Segmentation, grid-level aggregation, and prediction points

**Fig. 1:**
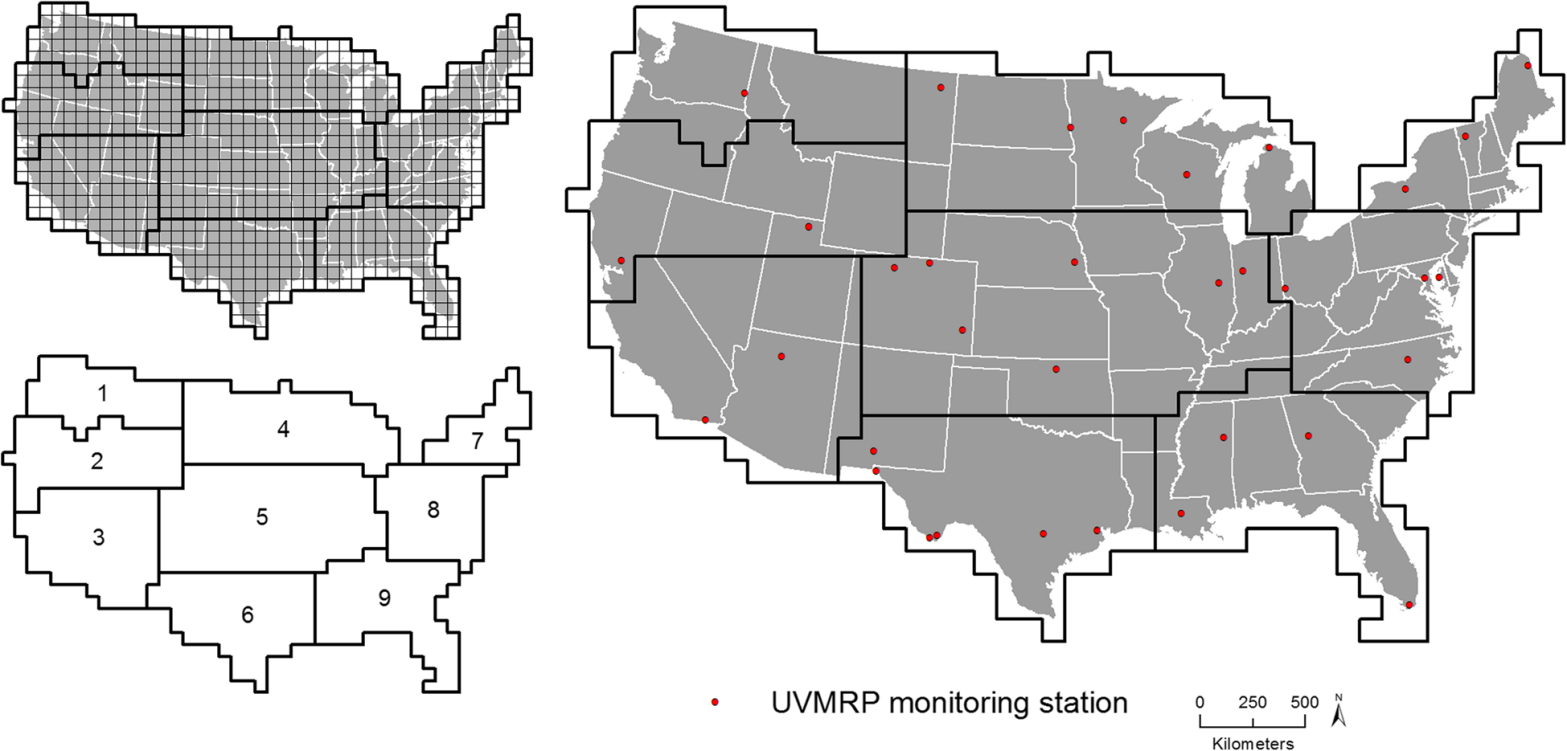
Regions used for ATP residual kriging and UVMRP monitoring stations. *Top left*: Nine regions created using segmentation within which ATP residual kriging was conducted. *Bottom left*: Region 1, northwest; 2, Pacific mid-west; 3, southwest; 4, north central; 5, mid-central; 6, south central; 7, northeast; 8, mid-Atlantic; 9, southeast. Right: U.S. Department of Agriculture (USDA) UV-B Monitoring and Research Program (UVMRP) monitoring stations (*n* = 31) used as the gold standard in model validation

Segmentation was performed to create regions of relatively homogeneous UV_Ery_ across the contiguous U.S. within which to perform stratified kriging. A stratified kriging approach was implemented as previous studies have modeled within different strata/areas to satisfy the kriging assumption of stationarity [36], and to emulate the adaptive ATP residual kriging approach conducted in Wang et al. [30]. Specifically, a global regression model may not adequately address local variation in UV_Ery_, where the association between the predictors and UV_Ery_ may vary within a study area. The UV_Ery_ gridded raster from 1980 (any year would have been sufficient) was converted to a polygon layer with the polygon boundaries defined by the boundaries of each grid. The USGS state boundaries layer [51] was overlaid with the grid polygon layer to determine specific grids intersecting the U.S. Grids from the preprocessed UV_Ery_ rasters from July 1980, 1990, 2000, and 2010 within the U.S. boundary were input into the segmentation procedure. Segmentation, which uses a watershed delineation method of merging/growing pixels across input bands exhibiting minimal variance [52], was performed on the U.S. grids in IDRISI Selva using the following parameters: window of 3, tolerance of 1 to 10 using intervals of 1, weight mean factor of 0.5, and weight variance factor of 0.5. Based on a visual comparison of resultant segments (polygons) from each year with a priori knowledge of latitude-driven trends in UV_Ery_, nine regions were created for modeling (Fig. 1). Each grid was associated with one region. Separately within each region each year, all ancillary data were aggregated to the grid level to calculate a mean grid value (e.g., mean AOD within each grid in 1998). A 1 km fishnet grid across the contiguous U.S. was created to serve as the prediction points, which were spatially joined (intersected) with the polygon grid layer to determine which prediction point corresponded to which grid. In practice, at least eight points should discretize each area [53]. All preprocessed, non-aggregated ancillary data were spatially joined (intersected) to the prediction points for later calculation of downscaled UV_Ery_.

### Model development and covariate selection

Linear mixed-effects regression was performed separately for each region using SAS (SAS Institute, Cary, NC). Random intercepts for each grid accounted for repeated measures by biennial year from 1998 to 2012. We a priori determined that the following established predictors of UV_Ery_ would be included in all regional models: AOD, elevation, latitude, ozone, and year. Manual backward elimination (variable removal from model if *p* > 0.05) and examination of goodness-of-fit (R^2^) were used to select final models for each region. Candidate models were compared based on their ability to maximize R^2^, which was calculated using a published method for random intercept linear mixed-effect regression models as the proportional reduction in the estimated total residual variance comparing the null model without covariates with the model of interest [54, 55]. Variables whose removal resulted in ≥10% change in a predictor(s) effect estimate were kept in the model. Interactions between year and each predictor included in the final models were examined by including an interaction term in the model. Statistically significant interactions (*p* < 0.05), as well as interactions where examination of the year-specific effect estimates of a predictor showed relatively high variability, were included in the final models. Assumptions for linear mixed-effects regression models (linearity, homoscedasticity, and normality of residuals) were checked using plots, and non-independence is accounted for through mixed-effects models considering both between- and within-cluster variances.

### Stratified ATP residual kriging

#### Simple kriging

At each prediction point, a downscaled residual value was calculated using the following equation [24, 25, 37]:

$$ z\left({u}_n\right)={\displaystyle \sum_{i=1}^K}{w}_i\left({u}_n\right)z\left({v}_i\right) $$

where *z*(*u*_*n*_) is the downscaled residual value at prediction point *u*_*n*_ for the *n*th discretizing point, *K* is the number of grids neighboring the prediction point *u*_*n*_, and *w*_*i*_(*u*_*n*_) is the simple kriging weight assigned to the residual value *z*(*v*_*i*_) of grid (*v*_*i*_) for the *i*th grid neighboring the prediction point *u*_*n*_. The neighborhood was defined by the 16-nearest neighbors. Sensitivity analyses using the 8-nearest neighbors showed similar results (results not shown). The kriging weights were calculated by solving for the following simple kriging system [24, 26]:

$$ {\displaystyle \sum_{i=1}^K}{w}_i\left({u}_n\right)\overline{C}\left({v}_j,{v}_i\right)=\overline{C}\left({u}_n,{v}_j\right)\kern2em j=1,\dots, K, $$

where $\overline{C}\left({v}_j,{v}_i\right)$ is the ATA covariance and $\overline{C}\left({u}_n,{v}_j\right)$ is the ATP covariance for the *i*th and *j*th grids. As an example to illustrate ATA and ATP covariance, an ATA covariance matrix A is comprised of elements *A*_*ij*_ with row *i* and column *j*, where *i* and *j* correspond to the grids within each neighborhood. Element *A*_2,1_ is the average of the covariance values between any two points discretizing grids 1 and 2 within the neighborhood for a given prediction point. An ATP covariance vector b is comprised of elements *b*_*n*_ with *n* rows, where *n* corresponds to the grids neighboring the prediction point. Element *b*_1_ is the average of the covariance values between the prediction point and any point discretizing grid 1 within the neighborhood of the prediction point. ATA and ATP covariances are estimated using a point support variogram of the residuals, which is calculated from the grid-level (i.e., area-level) variogram of the residuals discussed in the next section. A variogram depicts the spatial structure or correlation of a phenomenon of interest, showing semivariance (mean of the squared differences of all pairs of points, e.g., grid centroids, a particular distance apart) vs. distance [56]. Covariance is calculated by subtracting the semivariance from the sill [57]. Kriging was performed in SpaceStat.

### Variogram deconvolution

Variogram deconvolution was used to estimate the point support variogram from the grid-level variogram through an iterative process in SpaceStat based on Goovaerts [37]. Variogram deconvolution determines the optimal point support variogram whose regularized (i.e., averaged) variogram most closely approximates the grid-level variogram. The grid-level variogram is first designated as the optimal point support variogram. A regularized variogram is calculated from the optimal point support variogram using the following equation [37, 53]:

$$ {\gamma}_v(h)=\gamma (h)-\overline{\gamma}\left(v,v\right) $$

where *γ*_*v*_(*h*) is the regularized variogram value at distance lag *h*, *γ*(*h*) is the point support variogram at distance lag *h* used in practice to approximate the ATA variogram value $\overline{\gamma}\left(v,{v}_h\right)$ (at very large distances where *h* > distance across the grid, the ATA variogram approximately equals the point variogram [58]), and $\overline{\gamma}\left(v,v\right)$ is the within-area (i.e., within-grid) variogram value. The within-area variance is calculated using the discretizing points and the optimal point support variogram. As all grids are the same size and shape in this study, the within-area variance is constant. A difference statistic is calculated as the average relative difference between the two curves of the regularized and grid-level variograms over the distance lags. A rescaled point support variogram is then calculated by multiplying the optimal point support variogram by a weight that incorporates information from the grid-level and regularized variogram. Rescaling serves to minimize the difference statistic. This process is performed iteratively until the difference statistic value is sufficiently small, the maximum number of iterations has been reached, or a small decrease in the difference statistic is recorded a given number of times.

### Prediction of downscaled UV_Ery_ and model validation

The downscaled UV_Ery_ value at each prediction point was calculated by adding the downscaled residual value to the final region-specific random intercept linear mixed-effects regression model. Model validation compared UV_Ery_ predicted using ATP residual kriging and values from the NASA TOMS and OMI grids to UV_Ery_ observed at U.S. Department of Agriculture (USDA) UV-B Monitoring and Research Program (UVMRP) monitoring stations across the U.S. (the gold standard) (Fig. 1). The UVMRP is a national network of stations designed to monitor and examine UV-B at the Earth’s surface and to study the interaction between UV-B radiation, agriculture, forests, ecosystems, and climate [59]. There were 31 monitoring stations in the contiguous U.S. used in this study (Fig. 1), each operational during different years (Additional file 1: Table S1). UV_Ery_ is calculated using UV-B irradiance acquired from broadband UVB-1 pyranometers weighted by the McKinlay and Diffey model [40, 59]. Mean absolute errors (MAEs) and root mean square errors (RMSEs) were calculated using UV_Ery_ predicted from ATP residual kriging or NASA grids and UV_Ery_ observed at the UVMRP monitoring stations. The percent relative improvement in MAE and RMSE was calculated by comparing ATP residual kriging to NASA grids.

### Supplemental analyses: coherence property and grid-level UV_Ery_ temporal variability

ATP residual kriging satisfies the coherence (i.e., pycnophylactic or mass-preserving) property, where the average of the predicted UV_Ery_ values of the prediction points discretizing a given NASA grid should equal the UV_Ery_ value of the NASA grid. Point-level predicted UV_Ery_ values within each NASA grid were averaged and compared to the NASA grid UV_Ery_ value using Spearman rank correlation coefficients. To examine temporal variation in inter-annual UV_Ery_, we compared average July NASA TOMS and OMI grid-level UV_Ery_ every year from 1998 to 2012 using repeated-measures analysis of variance (ANOVA). Yearly TOMS and OMI UV_Ery_ images were converted to point layers and intersected with the USGS state boundaries layer to determine inclusion into the analysis.

## Results

Final random intercept linear mixed-effects regression models for each region are shown in Additional file 1: Tables S2–S10. Between *n* = 38 and 155 grids were included in the models for each of the 8 years in the study time period from 1998 to 2012. Overall, directions of effect were consistent with previous research such as an inverse association between latitude and UV_Ery_. Several grid-level regression models were associated with low to moderate R^2^ values (e.g., Pacific mid-west R^2^ 0.53) (Additional file 1: Table S11). The point support variograms calculated from variogram deconvolution were characterized by higher sills compared to the associated grid-level variograms (the semivariance value after which points are no longer spatially correlated), which is expected as any areal datum (e.g., a NASA grid) is defined as the average of all point support values within the datum (data not shown).

**Fig. 2:**
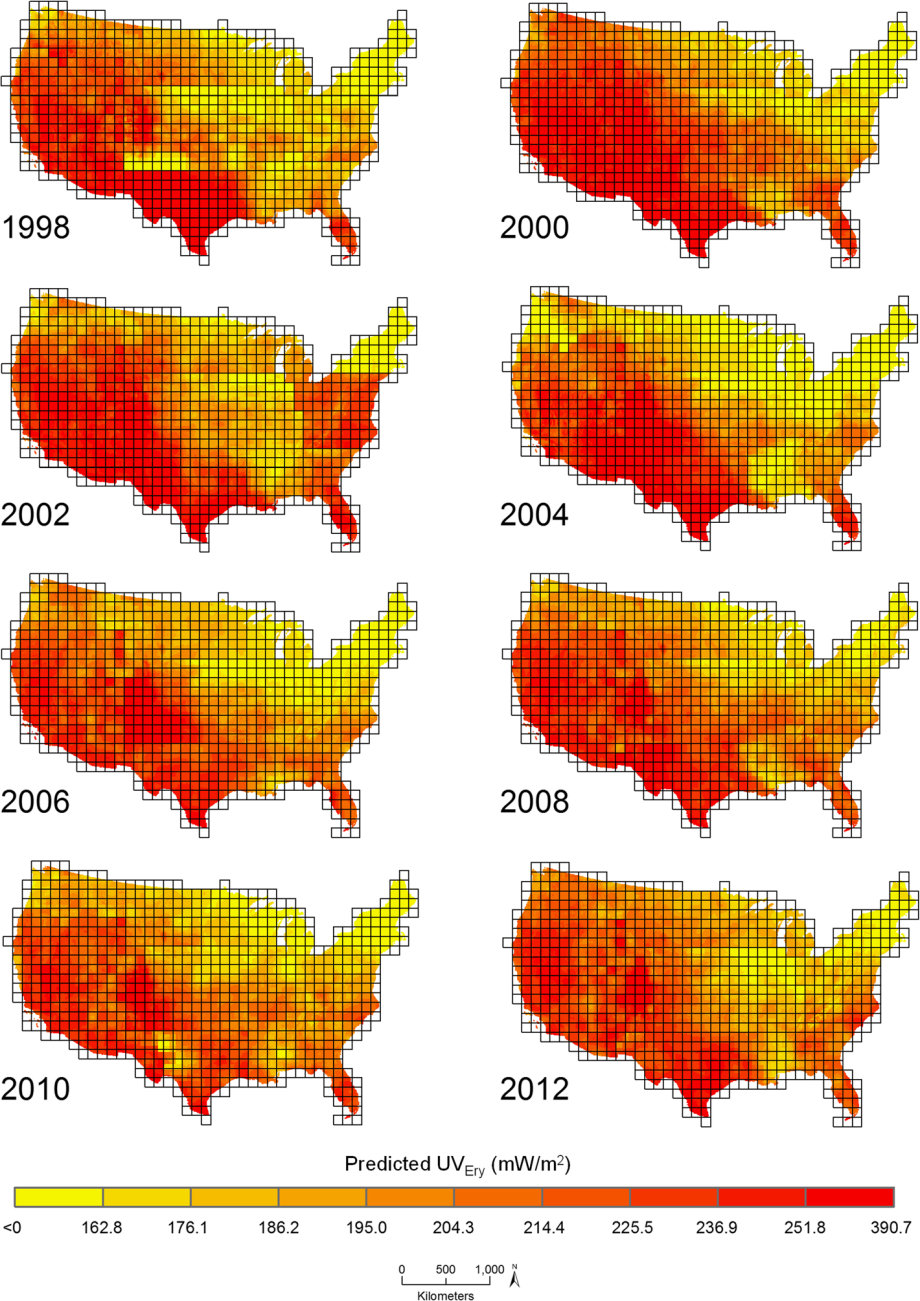
Downscaled average July UV_Ery_ from ATP residual kriging models in the contiguous U.S. (1998–2012)

**Fig. 3:**
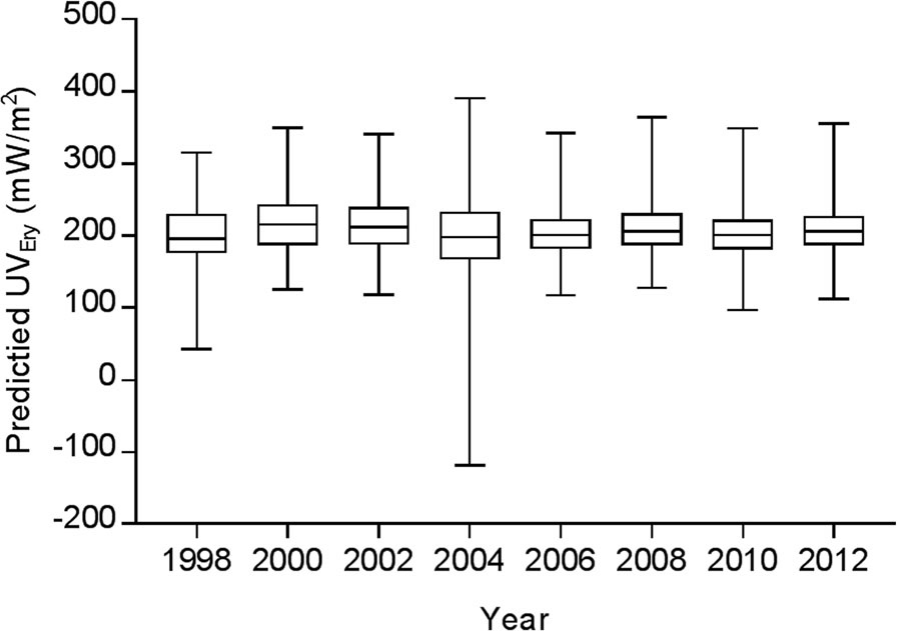
Boxplots of downscaled average July UV_Ery_ from ATP residual kriging in the contiguous U.S. (1998–2012)

**Fig. 4:**
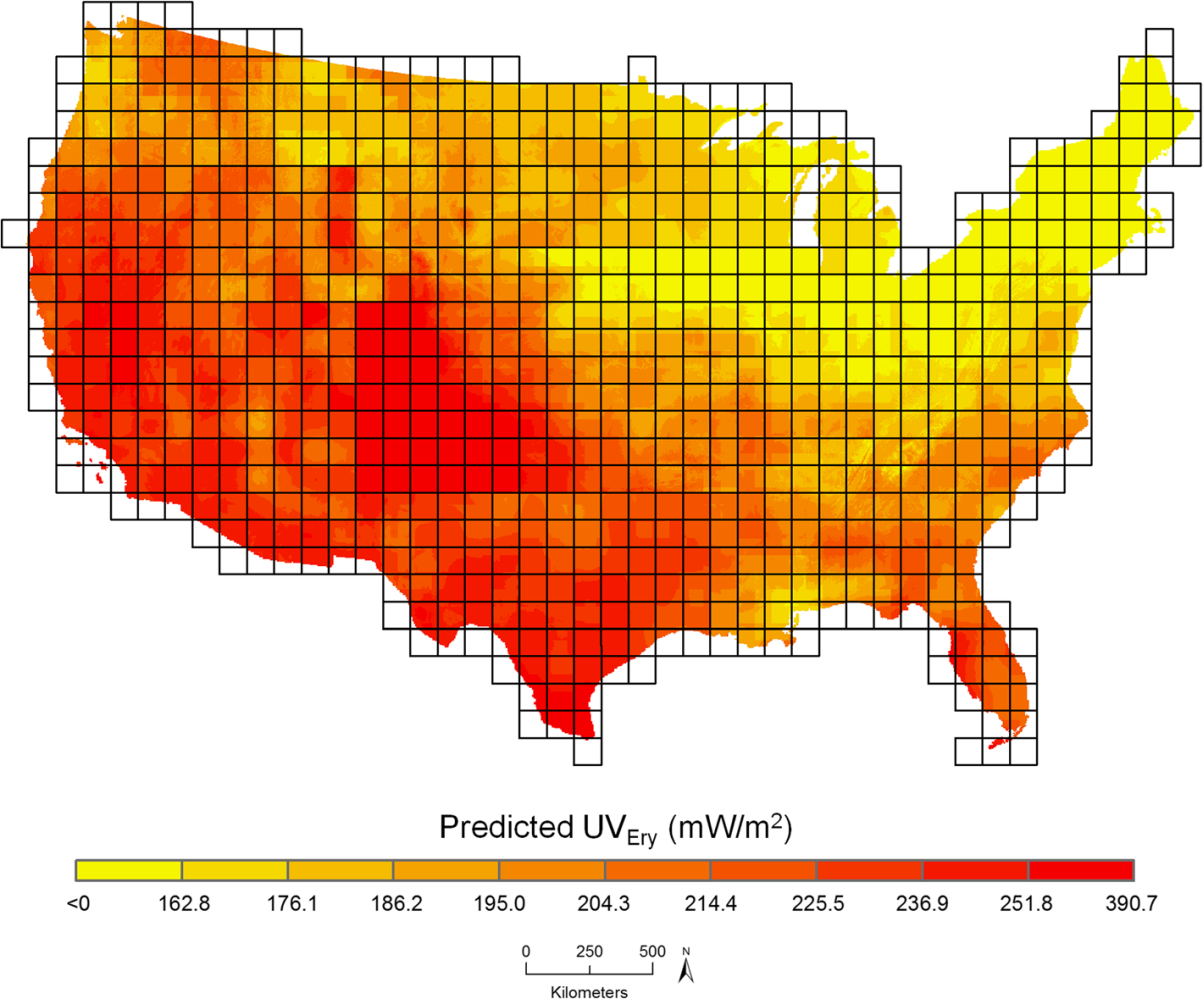
Downscaled average July UV_Ery_ from ATP residual kriging models in the contiguous U.S. in 2006

Figure 2 shows downscaled average July UV_Ery_ predicted from ATP residual kriging separately performed within nine regions across the U.S. biennially from 1998 to 2012. Predicted values ranged between <0 to 390.7 mW/m^2^ (Fig. 3). Temporal variability in UV_Ery_ can be observed in the different geographic distributions of predicted UV_Ery_ values across the U.S. year-to-year. As expected, there is a pattern of increasing UV_Ery_ values in each year with decreasing latitude moving south towards the Equator. A closer examination of 2006 (Fig. 4) allows for visualization of the spatial variability in predicted UV_Ery_ values within the original NASA grids, produced as a result of downscaling from the grid- to finer point-level spatial resolution. Table 2 shows results of the validation comparing UV_Ery_ predicted from ATP residual kriging or from NASA grids vs. UV_Ery_ observed at UVMRP monitoring stations (Additional file 1: Tables S12–S14 provide detailed validation results by UVMRP station). On average from 1998 to 2012, using ATP residual kriging was associated with a 22.0% relative improvement in MAE and a 16.8% relative improvement in RMSE compared to using NASA grids to predict UV_Ery_ observed at the UVMRP monitoring stations. Although the NASA grids provided more accurate UV_Ery_ estimates in 2010, MAE and RMSE values were relatively similar when using ATP residual kriging or NASA grids to predict UV_Ery_. There were also regional differences in model predictive performance, where ATP residual kriging provided positive percent relative improvements with respect to MAE and RMSE in each region (Table 3). The largest percent relative improvement in using ATP residual kriging vs. NASA grids to predict UV_Ery_ at the UVMRP monitoring stations was observed in the southeast, while the lowest (positive) percent relative improvement was observed in the mid-Atlantic.

**Table 2:** Validation of UV_Ery_ from ATPRK vs. NASA grids using UVMRP stations by year (1998–2012)

				% relative improvement	
Year	Prediction model	MAE	RMSE	MAE	RMSE
1998	NASA	17.4	20.1	--	--
	ATPRK	13.7	16.6	21.6	17.5
2000	NASA	26.5	30.9	--	--
	ATPRK	13.8	17.9	48.1	42.0
2002	NASA	26.9	31.2	--	--
	ATPRK	19.2	28.3	28.5	9.3
2004	NASA	23.5	31.0	--	--
	ATPRK	13.9	19.2	40.6	38.1
2006	NASA	15.4	20.4	--	--
	ATPRK	13.8	19.3	10.4	5.2
2008	NASA	17.2	20.7	--	--
	ATPRK	12.7	16.2	26.2	21.6
2010	NASA	13.9	18.8	--	--
	ATPRK	17.2	23.7	−23.8	−26.4
2012	NASA	21.7	31.3	--	--
	ATPRK	22.4	28.7	−3.4	8.1
Average 1998–2012	NASA	20.3	25.5	--	--
	ATPRK	15.8	21.3	22.0	16.8

Abbreviations: *ATPRK* area-to-point residual kriging, *MAE* mean absolute error, *NASA* National Aeronautics and Space Administration, *RMSE* root mean square error

**Table 3:** Validation of UV_Ery_ from ATPRK vs. NASA grids using UVMRP stations by region (1998–2012)

				% relative improvement	
Region	Prediction model	MAE	RMSE	MAE	RMSE
Northwest	NASA	10.8	10.8	--	--
	ATPRK	9.9	9.9	8.7	8.7
Pacific mid-west	NASA	15.6	16.8	--	--
	ATPRK	11.6	12.2	25.5	27.4
Southwest	NASA	23.6	26.4	--	--
	ATPRK	18.0	22.4	23.8	15.3
North central	NASA	13.4	15.1	--	--
	ATPRK	9.8	11.5	27.4	23.7
Mid-central	NASA	21.3	23.7	--	--
	ATPRK	17.2	20.0	19.3	15.8
South central	NASA	19.6	22.9	--	--
	ATPRK	16.7	19.4	14.6	15.5
Northeast	NASA	17.4	19.2	--	--
	ATPRK	13.9	16.5	19.7	14.1
Mid-Atlantic	NASA	14.9	16.4	--	--
	ATPRK	14.8	15.8	0.6	3.6
Southeast	NASA	36.9	43.3	--	--
	ATPRK	25.3	30.6	31.5	29.4

Abbreviations: *ATPRK* area-to-point residual kriging, *MAE* mean absolute error, *NASA* National Aeronautics and Space Administration, *RMSE* root mean square error

The coherence property was satisfied in each year, demonstrated by the very strong correlations between the original NASA UV_Ery_ grid values and the average of the predicted UV_Ery_ values at points discretizing each grid (r_s_ ranging between 0.82 and 0.93) (Additional file 1: Table S15). At the regional level, the coherence property was satisfied in the mid-Atlantic, mid-central, north central, Pacific mid-west, south central, and southwest (Additional file 1: Table S16). Analysis of the temporal variation in inter-annual UV_Ery_ showed a statistically significant difference in NASA grid-level average July UV_Ery_ values across the U.S. each year from 1998 to 2012 (*p* < 0.0001) (*n* = 782 grids intersecting the contiguous U.S.). Yearly median NASA grid-level average July UV_Ery_ values ranged between 214.5 and 237.1 mW/m^2^ (Additional file 1: Table S17).

## Discussion

ATP residual kriging was used to create a validated spatially and temporally varying exposure model of average July UV_Ery_ across the contiguous U.S biennially from 1998 to 2012. A stratified kriging approach was conducted to build separate random intercept linear mixed-effects regression models within nine U.S. regions. Predictors in the final regional models included AOD, cloud cover, elevation, latitude, ozone, year, as well as interactions such as between year and AOD. The validation, through comparing MAE and RMSE, showed that on average, UV_Ery_ estimated using ATP residual kriging more accurately predicted UV_Ery_ observed at USDA UVMRP monitoring stations compared to using NASA grids. Usage of ATP residual kriging also provided positive percent relative improvements with respect to MAE and RMSE compared to using NASA grids within each of the nine U.S. regions. To the best of our knowledge, this is the first application of ATP residual kriging to UV exposure modeling.

ATP residual kriging is considered a relatively new method that has been thus far used to downscale imagery and environmental variables [30]. Several important considerations in modeling UV_Ery_ were given careful attention in this study. In particular, it was essential to create a time-varying exposure model to address climate change and related issues that have and will continue to have an impact on the amount of UV reaching the Earth’s surface. Although authors of an existing UV model noted no statistically significant difference in GHI measured between three 10-year intervals from 1961 to 1990, surface UV radiation has been observed to exhibit high variability [11], especially given changes in ozone layer depletion over time [33]. The most important determinant of surface UV-B is ozone, a greenhouse gas that absorbs UV and is subject to high year-to-year variability due to variation in atmospheric circulation [60]. There were observed decreases in ozone between the 1960s and 1990s, although there is evidence to suggest that the global ozone layer is beginning to recover since 2000 [60]. Furthermore, climate change due to increasing concentrations of greenhouse gases and variability in UV-absorbing tropospheric gases, aerosols, and clouds may also have indirect impacts on surface UV. Increasing greenhouse gases will increase large-scale transport and overturning of the upper atmosphere, which is predicted to lead to increases in ozone outside of the tropics. Importantly, in our study, we observed a statistically significant difference in average July UV_Ery_ each year from 1998 to 2012 using NASA grids, further demonstrating the need to account for temporal variability in modeling UV_Ery_.

The current model improves on the spatial and spectral resolution of previous models. Our model’s target scale was 1 km, providing greater spatial resolution in UV estimates compared to the NASA TOMS and OMI grids as well as the state level [10]. In addition, rather than modeling UV exposure across all wavelengths, we specifically modeled UV_Ery_, internationally recognized as the preferred method of reporting UV-B exposure, especially as different studies have used different wavelength ranges to define UV-B [61]. We were also able to incorporate other important predictors of UV_Ery_ in model creation beyond the variables used in previous exposure models (e.g., AOD and ozone), which improved our model’s predictive performance by explaining more of the variance in UV_Ery_.

It is important to note that although UV_Ery_ has been used in previous epidemiologic studies addressing vitamin D-related research questions [14–17, 19, 20, 22], vitamin D-weighted UV, calculated using the MacLaughlin et al. [62] CIE action spectrum for previtamin D_3_ synthesis in human skin, is a method that specifically considers the effectiveness of UV wavelengths in producing previtamin D_3_ (optimum wavelengths 295–300 nm). The relevance of UV_Ery_ to previtamin D_3_ synthesis is also tied to the wavelengths beyond 295–300 nm included in the UV_Ery_ calculation, and the extent to which these wavelengths are affected by factors such as solar zenith angle (SZA) and altitude. UV radiation is attenuated at lower SZAs due to the longer distance photons have to travel through the ozone layer, increasing the probability of absorption [63]. At higher altitudes, UV radiation is higher as there is a shorter path through which UV travels and less scattering molecules above elevated surfaces [2, 64].

Our UV_Ery_ exposure model can be applied to exposure assessment in epidemiologic studies. UV_Ery_ is a biologically relevant exposure that has been used in previous epidemiologic studies examining human health outcomes [14–20], addressing research questions related to DNA damage [21] and the effects of vitamin D [14–17, 19, 20, 22]. Updated geocoded residential locations can be overlaid with this predicted UV_Ery_ surface to calculate time-varying exposures, and can be used as a proxy for vitamin D status along with other information. In particular, our UV_Ery_ spatiotemporal exposure model represents an improvement over using surrogate measures such as latitude and season. Lifetime vitamin D status is ideally captured through a combination of baseline blood 25-hydroxyvitamin D concentrations and questionnaires collecting lifetime dietary, supplemental, and sunlight exposure [65]. Serum 25-hydroxyvitamin D is used for short-term vitamin D status, while long-term dietary and supplemental intake and sunlight exposure is most feasible for lifetime vitamin D assessment. Latitude and season have been used as proxies for vitamin D exposure as the majority of vitamin D is produced from the skin’s synthesis after solar UV-B exposure. However, maps depicting our validated UV_Ery_ exposure model in this study show variability of predicted UV_Ery_ within a given latitude. The presented ATP residual kriging UV_Ery_ exposure model can be used to reduce measurement error in geospatial proxies for vitamin D exposure, providing a valuable means to measure ambient UV_Ery_ levels relevant to UV-B exposure. Additional individual-level information should also be ascertained, including work location, time spent indoors, skin pigmentation, sunscreen use, amount of skin exposed, and clothing type [66, 67].

The final random intercept linear mixed-effects regression models selected for each region differed according to which variables were included and often times the direction and magnitude of the associations between the predictors and UV_Ery_. Apart from AOD, elevation, latitude, ozone, and year, which were a priori included in each regional model, variables selected for inclusion into final models included surface albedo, cloud cover, dew point, SIS, and SO_2_. Several interactions with year were included in the models, including with surface albedo, AOD, cloud cover, dew point, elevation, SO_2_, and latitude. Different geographic regions across the U.S. are characterized by different climates, and thus different physical, meteorological, and climatological variables may exert varying impacts on surface UV_Ery_ at different locations and at different points in time [68]. For example, total ozone is not uniformly distributed across the Earth, but rather varies with latitude, season, and natural air motions [69]. AOD as well as UV-absorbing aerosols including SO_2_ exhibit spatial and temporal variations across the globe, especially as air pollution is associated with large urban centers [70–72]. Thus, allowing for varying regional models that include different predictors and interactions with time directly addresses the adaptive ATP residual kriging methods employed in a previous study and accounts for the potential local-level relationships between the predictors and UV_Ery_ [30].

Overall, the directions of effect associated with the predictors included in the final models were expected (e.g., inverse associations between UV and AOD, latitude, and ozone). However, apart from the models where a priori-determined variables were included (e.g., ozone and elevation) irrespective of statistical significance and where the main effects were accompanied by an interaction term with year, there were several models characterized by unexpected associations. For example, regional models for the mid-central, northeast, south central, and southeast showed statistically significant inverse associations between surface albedo and UV_Ery_. Surfaces can return radiation up towards the atmosphere, which is partially scattered back to the ground [2]. It is possible that differences in surface albedo as well as surface UV in urban vs. rural areas within these four regions may be driving this inverse association. For example, urban areas are generally characterized by higher surface albedos compared to rural areas [73]. If there are positive associations between surface albedo and UV in both rural and urban areas, but UV values observed in urban areas are relatively lower compared to rural areas (due to differences in reflectivity of incident irradiance on non-horizontal surfaces, e.g., buildings [74]), this would result in a negative association. The extent to which rural- and urban-specific surface albedos should be considered in modeling should be explored [75]. In the southeast, there was a statistically significant inverse relationship between elevation and UV_Ery_. Higher elevations are generally associated with less scattering aerosols, and thus higher UV [2]. However, the southeast is a coastal region that is largely characterized by low elevations at or below sea level [76], which as a result of dominating the landscape, may have driven the inverse association. It is possible that aggregation of the ancillary data to the grid level resulted in unexpected associations related to the modifiable areal unit problem (MAUP), or different observed patterns and relationships based on how data are aggregated [56]. However, as the validation demonstrated, despite these unexpected associations, ATP residual kriging was still able to achieve more accurate predictions of UV_Ery_ at UVMRP monitoring locations compared to using NASA grids, and coherence was achieved across all years.

Strengths of this study include the high spatial and temporal resolution of the UV_Ery_ exposure model compared to several previous models. In particular, our model’s 1 km spatial resolution is finer than the Scotto et al. [10] state-based model. The results of the validation showed that UV_Ery_ exhibits spatial variability within the NASA grids, which are generally smaller in size than most states. We created a time-varying exposure model with predictions for each year (biennially) from 1998 to 2012, which is an improvement over previous models that aggregated UV exposure values over many years [10, 11]. This method can be readily adapted to historical exposure assessment, important to consider in studying chronic health outcomes associated with latency periods. We were able to include many important predictors of UV_Ery_ that have not been considered in previous UV exposure modeling efforts such as AOD and ozone. We also incorporated the paradigm of considering local-level variation in ATP residual kriging pioneered in a recent study [30]. We implemented a stratified kriging approach, which did not rely on a single global regression model to describe the relationship between the predictors and UV_Ery_, but rather addressed potential regional differences in their relationships.

Limitations include the reliance of ATP residual kriging on the spatial resolution of the ancillary data. Although all ancillary data were of a finer spatial resolution compared to the NASA grids being downscaled, pixel sizes ranged between 0.1 and 3500 km^2^. The spatial resolution of our ATP residual kriging model is 1 km (downscaled from approximately 100 × 100 km NASA grids), which improves on several coarser resolution models, but significant variation in UV_Ery_ within a 1 km pixel may still exist. Use of discretization geography finer than the 1 km level should be explored. Although we did not adjust for the UV predictors of snow cover and SZA, we did consider surface albedo (snow is associated with high surface albedo), and accounted for latitude while holding constant time of day (noon-time) and season (summer) in our modeling, the three determinants of SZA [2]. There were relatively few UVMRP monitoring stations available for the validation. As these stations were designed to be located in more rural areas to address the agricultural aims of the UVMRP, and the purpose of this model is for use in epidemiologic exposure assessment, it would be informative to understand the extent to which the ATP residual kriging model predicts UV in more highly populated non-rural areas [77]. NASA achieved better model predictive performance in 2010 with respect to MAE and RMSE, and in 2012 with respect to MAE. It should be noted that 2010 has been described as an aberrant year associated with unusually high values of total ozone [60]. However, despite the lack of improvement in using ATP residual kriging to predict UV_Ery_ in these years, the absolute differences in MAE and RMSE between the two models are not substantially large. As the NASA grids are a major input into the ATP residual kriging process, specifically used as the outcome variable in creating the regression models, it would be expected that if ATP residual kriging does not improve prediction, it would provide results similar to when using the original source units. Although the majority of regions and all years achieved coherence, predicted point-level UV_Ery_ values were not highly correlated with original NASA grid UV_Ery_ values in the southeast, northeast, and northwest. The stratified modeling approach is also subject to edge effects as we imposed artificial boundaries within which to conduct modeling for the purposes of achieving stationarity and addressing local-level variation in predictor-UV_Ery_ relationships [56]. Points near the edges of each region may have neighboring grids outside of the region that were not considered when calculating the kriging weights. Future modeling efforts could include grids and prediction points outside of but adjacent to a given region during interpolation, and subsequently clip the prediction surface to the original region [78]. Finally, this method is time- and resource-intensive, especially due to applying variogram deconvolution and simple kriging separately for each year to create a temporally varying exposure model. Researchers should consider the computational burden of conducting ATP residual kriging, minimizing the study area size, and/or determining if fewer time points can be included.

## Conclusions

ATP residual kriging was used to create a validated 1 km resolution spatiotemporal exposure model of average July UV_Ery_ across the contiguous U.S. biennially from 1998 to 2012. On average, ATP residual kriging was able to more accurately predict UV_Ery_ observed at USDA UVMRP monitoring stations compared to using coarser NASA grids. To the best of our knowledge, this study represents the first application of this method to exposure modeling of UV, adding to a growing body of literature modeling environmental variables using ATP residual kriging. This spatially and temporally varying UV_Ery_ exposure model can be used in individual-level exposure assessment to conduct epidemiologic studies clarifying the role ambient UV-B may play in human health outcomes.

## Additional file

Additional file 1: Supplemental tables for UVMRP monitoring stations, regional linear mixed-effects regression models, model goodness-of-fit, validation results by UVMRP station, coherence property analysis, and yearly NASA grid-level July UV_Ery_ descriptive statistics. (DOCX 81 kb)

## Abbreviations

3DEP: 3D elevation program
ADAS: Atmospheric data assimilation system
ANOVA: Analysis of variance
AOD: Aerosol optical depth
ATA: Area-to-area
ATP: Area-to-point
ATPRK: Area-to-point residual kriging
CIE: Commission Internationale de l’Eclairage
DNA: Deoxyribonucleic acid
GHI: Global horizontal irradiance
GIS: Geographic information system
GOES-5: Goddard earth observing system model, version 5
IARC: International agency for research on cancer
IQR: Interquartile range
MAE: Mean absolute error
MAUP: Modifiable areal unit problem
MERRA: Modern era retrospective analysis for research and applications
MODIS: Moderate resolution imaging spectroradiometer
NASA: National Aeronautics and Space Administration
OMI: Ozone monitoring instrument
PRISM: Parameter elevation regression on independent slopes model
RMSE: Root mean square error
SD: Standard deviation
SIS: Surface incoming shortwave flux
SO_2_: Sulfur dioxide
SZA: Solar zenith angle
TOMS: Total ozone mapping spectrometer
USDA: United States Department of Agriculture
USGS: United States Geological Survey
UV: Ultraviolet radiation
UV-A: Ultraviolet A radiation
UV-B: Ultraviolet B radiation
UV-C: Ultraviolet C radiation
UV_Ery_: Erythemal ultraviolet radiation
UVMRP: UV-B monitoring and research program
WCS: Web coverage service
