# Supplementary material for: Spatiotemporal exposure modeling of ambient erythemal ultraviolet radiation
Source: Environ Health. 2016 Nov 24;15:111. doi: 10.1186/s12940-016-0197-x (PMC5121956; doi:10.1186/s12940-016-0197-x)
Supplement: Additional file 1: — Supplemental tables for UVMRP monitoring stations, regional linear mixed-effects regression models, model goodness-of-fit, validation results by UVMRP station, coherence property analysis, and yearly NASA grid-level July UVEry descriptive statistics. (DOCX 81 kb) [file 12940_2016_197_MOESM1_ESM.docx]

**Additional File 1** Supplemental tables for UVMRP monitoring stations, regional linear mixed-effects regression models, model goodness-of-fit, validation results by UVMRP station, coherence property analysis, and yearly NASA grid-level July UV_Ery_ descriptive statistics

**Table 1** UVMRP stations used for ATP residual kriging model validation in the contiguous U.S. (1998-2012)

|  | State | City | Station name | Date opened | Years contributed to validation^a^ | Station code |
| --- | --- | --- | --- | --- | --- | --- |
| 1 | Arizona | Flagstaff | Abyss Site at Grand Canyon National Park | 3/14/1996 | 1998-2012 | AZ01 |
| 2 | California | Davis | UC Davis Climate Station | 6/29/1994 | 1998-2012 | CA01 |
| 3 | California | Holtville | UC Desert Research and Extension Center | 2/18/1998 | 1998-2012 | CA21 |
| 4 | Colorado | Nunn | Central Plains Experimental Range | 1/30/1994 | 1998-2012 | CO01 |
| 5 | Colorado | Steamboat Springs | DRI Storm Peak Lab (roof) | 3/18/1999 | 2000-2010 | CO11 |
| 6 | Colorado | Lamar | Ultra-High Energy Cosmic Rays research (Auger Project) | 12/17/2003 | 2006-2012 | CO41 |
| 7 | Florida | Homestead | Beard Research Center at Everglades National Park | 5/5/1998 | 1998-2012 | FL01 |
| 8 | Georgia | Griffin | University of Georgia-Griffin Campus-Bledsoe Research Farm | 6/7/1994 | 1998-2012 | GA01 |
| 9 | Illinois | Bondville | Bondville Environmental and Atmospheric Research Site | 8/6/1993 | 1998-2012 | IL01 |
| 10 | Indiana | West Lafayette | Agronomy Center for Research and Education | 4/7/2001 | 2002-2012 | IN01 |
| 11 | Louisiana | Baton Rouge | LSU Central Research Station | 2/9/1996 | 1998-2012 | LA01 |
| 12 | Maine | Presque Isle | Northern Maine Regional Office | 10/27/1995 | 1998-2012 | ME11 |
| 13 | Maryland | Queenstown | Wye Research and Education Center | 1/30/1996 | 1998-2012 | MD01 |
| 14 | Maryland | Beltsville | USDA Beltsville Agricultural Research Center - South Farm | 5/30/1999 | 2000-2012 | MD11 |
| 15 | Michigan | Pellston | University of Michigan Biological Station at Douglas Lake | 8/2/1994 | 1998-2012 | MI01 |
| 16 | Minnesota | Grand Rapids | North Central Research and Outreach Center | 11/13/1996 | 1998-2012 | MN01 |
| 17 | Mississippi | Starkville | Mississippi Agricultural and Forestry Experiment Station | 9/13/2000 | 2002-2012 | MS01 |
| 18 | Montana | Poplar | Fort Peck Assiniboine and Sioux Tribes | 9/30/1997 | 1998-2000, 2004-2012 | MT01 |
| 19 | Nebraska | Mead | High Plains Regional Climate Center | 5/16/1996 | 1998-2012 | NE01 |
| 20 | New Mexico | Las Cruces | Jornada Experimental Range | 9/22/1994 | 1998-2012 | NM01 |
| 21 | New York | Geneva | New York State Agricultural Experiment Station at Geneva | 8/5/1994 | 1998-2012 | NY01 |
| 22 | North Carolina | Raleigh | NCSU Air Quality Educational Unit | 10/3/2002 | 2004-2012 | NC01 |
| 23 | North Dakota | Fargo | NDSU Microclimatic Research Station | 10/29/2004 | 2006-2012 | ND01 |
| 24 | Ohio | Oxford | Ecology Research Center | 10/6/1993 | 1998-2000 | OH01 |
| 25 | Oklahoma | Billings | US Department of Energy ARM/SGP/CART | 2/17/1999 | 2000-2012 | OK01 |
| 26 | Texas | Panther Junction | Castolon Site at Big Bend National Park | 12/28/1996 | 1998-2008 | TX01 |
| 27 | Texas | Seguin | Texas Lutheran University (roof) | 3/16/2004 | 2004-2012 | TX21 |
| 28 | Texas | Houston | University of Houston - north Moody Tower (roof) | 8/3/2006 | 2008-2012 | TX41 |
| 29 | Utah | Logan | Utah Climate Center | 12/15/1995 | 1998-2012 | UT01 |
| 30 | Vermont | Burlington | Proctor Maple Research Center | 10/22/1996 | 1998-2012 | VT01 |
| 31 | Washington | Pullman | Palouse Conservation Field Station | 7/13/1994 | 1998-2012 | WA01 |
| 32 | Wisconsin | Dancy | Lake Dubay | 5/20/1996 | 1998-2012 | WI01 |

Note: This information was provided by the UV-B Monitoring and Research Program (UVMRP) (<http://uvb.nrel.colostate.edu/UVB/index.jsf>). These stations include those within the contiguous USGS U.S. states boundary layer with at least one year of non-missing data in July during one of the study years (biennially from 1998-2012).

^a^Modeling was performed biennially during even years.

**Table 2** Final grid-level random intercept linear mixed-effects regression model for the northwest

| Variable | Beta | p-value |
| --- | --- | --- |
| Intercept | 760.190 | <0.0001 |
| Surface albedo | 84.909 | 0.0062 |
| AOD | 136.060 | 0.1424 |
| Cloud cover | Not included | -- |
| Dew point | -0.395 | 0.0267 |
| Elevation | 0.015 | <0.0001 |
| Latitude | -0.040 | <0.0001 |
| Ozone | -1.434 | <0.0001 |
| SIS | Not included | -- |
| SO_2_ | Not included | -- |
| Year |  | <0.0001 |
| 1998 | Referent | -- |
| 2000 | 39.257 | 0.0004 |
| 2002 | 31.935 | 0.0038 |
| 2004 | 45.769 | 0.0002 |
| 2006 | 47.818 | <0.0001 |
| 2008 | 53.429 | <0.0001 |
| 2010 | 77.960 | <0.0001 |
| 2012 | 55.581 | <0.0001 |
| Year * AOD |  | 0.0002 |
| Year (1998) * AOD | Referent | -- |
| Year (2000) * AOD | -313.240 | 0.0018 |
| Year (2002) * AOD | -215.330 | 0.0282 |
| Year (2004) * AOD | -294.350 | 0.0047 |
| Year (2006) * AOD | -338.130 | 0.0007 |
| Year (2008) * AOD | -333.210 | 0.0007 |
| Year (2010) * AOD | -574.220 | <0.0001 |
| Year (2012) * AOD | -360.540 | 0.0003 |
| Year * elevation |  | <0.0001 |
| Year (1998) * elevation | Referent | -- |
| Year (2000) * elevation | -0.011 | <0.0001 |
| Year (2002) * elevation | -0.011 | <0.0001 |
| Year (2004) * elevation | -0.013 | <0.0001 |
| Year (2006) * elevation | -0.021 | <0.0001 |
| Year (2008) * elevation | -0.020 | <0.0001 |
| Year (2010) * elevation | -0.022 | <0.0001 |
| Year (2012) * elevation | -0.027 | <0.0001 |

Abbreviations: AOD, aerosol optical depth; SIS, surface incoming shortwave flux; SO_2_, sulfur dioxide.

Note: A total of n=480 observations (n=60 grids for 8 years from 1998-2012) contributed to this analysis. Refer to Table 1 for variable units.

**Table 3** Final grid-level random intercept linear mixed-effects regression model for the Pacific mid-west

| Variable | Beta | p-value |
| --- | --- | --- |
| Intercept | 524.920 | <0.0001 |
| Surface albedo | Not included | -- |
| AOD | -31.522 | 0.0105 |
| Cloud cover | Not included | -- |
| Dew point | -0.086 | 0.6421 |
| Elevation | 0.010 | 0.0004 |
| Latitude | -0.030 | <0.0001 |
| Ozone | -0.741 | <0.0001 |
| SIS | Not included | -- |
| SO_2_ | Not included | -- |
| Year |  | <0.0001 |
| 1998 | Referent | -- |
| 2000 | 32.176 | 0.0131 |
| 2002 | 22.692 | 0.1300 |
| 2004 | 40.313 | 0.0082 |
| 2006 | 56.056 | 0.0029 |
| 2008 | 59.881 | <0.0001 |
| 2010 | 55.428 | <0.0001 |
| 2012 | 58.967 | <0.0001 |
| Year * dew point |  | <0.0001 |
| Year (1998) * dew point | Referent | -- |
| Year (2000) * dew point | -0.605 | 0.0090 |
| Year (2002) * dew point | -0.332 | 0.2248 |
| Year (2004) * dew point | -0.636 | 0.0198 |
| Year (2006) * dew point | -0.938 | 0.0069 |
| Year (2008) * dew point | -0.997 | 0.0001 |
| Year (2010) * dew point | -1.015 | <0.0001 |
| Year (2012) * dew point | -1.093 | <0.0001 |
| Year * elevation |  | <0.0001 |
| Year (1998) * elevation | Referent | -- |
| Year (2000) * elevation | -0.006 | 0.0196 |
| Year (2002) * elevation | -0.003 | 0.2249 |
| Year (2004) * elevation | -0.009 | 0.0010 |
| Year (2006) * elevation | -0.019 | <0.0001 |
| Year (2008) * elevation | -0.016 | <0.0001 |
| Year (2010) * elevation | -0.015 | <0.0001 |
| Year (2012) * elevation | -0.020 | <0.0001 |

Abbreviations: AOD, aerosol optical depth; SIS, surface incoming shortwave flux; SO_2_, sulfur dioxide.

Note: A total of n=640 observations (n=80 grids for 8 years from 1998-2012) contributed to this analysis. Refer to Table 1 for variable units.

**Table 4** Final grid-level random intercept linear mixed-effects regression model for the southwest

| Variable | Beta | p-value |
| --- | --- | --- |
| Intercept | 294.620 | 0.0002 |
| Surface albedo | 225.740 | <0.0001 |
| AOD | -28.586 | 0.2025 |
| Cloud cover | Not included | -- |
| Dew point | -0.722 | 0.0028 |
| Elevation | 0.009 | 0.0051 |
| Latitude | -0.032 | <0.0001 |
| Ozone | -0.0003 | 0.9989 |
| SIS | Not included | -- |
| SO_2_ | 2622759 | 0.0001 |
| Year |  | 0.6178 |
| 1998 | Referent | -- |
| 2000 | -4.283 | 0.8427 |
| 2002 | 21.247 | 0.4256 |
| 2004 | -2.164 | 0.9246 |
| 2006 | -43.422 | 0.1152 |
| 2008 | -26.716 | 0.2773 |
| 2010 | -1.826 | 0.9368 |
| 2012 | -5.796 | 0.8070 |
| Year * surface albedo |  | <0.0001 |
| Year (1998) * surface albedo | Referent | -- |
| Year (2000) * surface albedo | -87.602 | 0.0008 |
| Year (2002) * surface albedo | -83.662 | 0.0015 |
| Year (2004) * surface albedo | -63.855 | 0.0184 |
| Year (2006) * surface albedo | -141.460 | <0.0001 |
| Year (2008) * surface albedo | -82.364 | 0.0014 |
| Year (2010) * surface albedo | -51.641 | 0.0508 |
| Year (2012) * surface albedo | -155.820 | <0.0001 |
| Year * dew point |  | 0.7007 |
| Year (1998) * dew point | Referent | -- |
| Year (2000) * dew point | 0.135 | 0.6214 |
| Year (2002) * dew point | -0.448 | 0.2053 |
| Year (2004) * dew point | -0.072 | 0.8223 |
| Year (2006) * dew point | 0.480 | 0.1798 |
| Year (2008) * dew point | 0.017 | 0.9535 |
| Year (2010) * dew point | -0.287 | 0.2983 |
| Year (2012) * dew point | -0.432 | 0.1275 |
| Year * elevation |  | 0.0002 |
| Year (1998) * elevation | Referent | -- |
| Year (2000) * elevation | 0.001 | 0.6899 |
| Year (2002) * elevation | -0.003 | 0.3664 |
| Year (2004) * elevation | 0.006 | 0.0490 |
| Year (2006) * elevation | -0.015 | <0.0001 |
| Year (2008) * elevation | -0.016 | <0.0001 |
| Year (2010) * elevation | -0.013 | <0.0001 |
| Year (2012) * elevation | -0.012 | <0.0001 |
| Year * SO2 |  | 0.2383 |
| Year (1998) * SO_2_ | Referent | -- |
| Year (2000) * SO_2_ | 761793.000 | 0.2801 |
| Year (2002) * SO_2_ | 1214513.000 | 0.0998 |
| Year (2004) * SO_2_ | 1581303.000 | 0.0345 |
| Year (2006) * SO_2_ | -1528054.000 | 0.0352 |
| Year (2008) * SO_2_ | -323111.000 | 0.6889 |
| Year (2010) * SO_2_ | 567876.000 | 0.4977 |
| Year (2012) * SO_2_ | 2132600.000 | 0.0120 |
| Year * latitude |  | 0.0002 |
| Year (1998) * latitude | Referent | -- |
| Year (2000) * latitude | 0.009 | 0.1214 |
| Year (2002) * latitude | 0.010 | 0.1045 |
| Year (2004) * latitude | 0.001 | 0.8031 |
| Year (2006) * latitude | 0.030 | <0.0001 |
| Year (2008) * latitude | 0.031 | <0.0001 |
| Year (2010) * latitude | 0.013 | 0.0281 |
| Year (2012) * latitude | 0.026 | 0.0002 |

Abbreviations: AOD, aerosol optical depth; SIS, surface incoming shortwave flux; SO_2_, sulfur dioxide.

Note: A total of n=752 observations (n=94 grids for 8 years from 1998-2012) contributed to this analysis. Refer to Table 1 for variable units.

**Table 5** Final grid-level random intercept linear mixed-effects regression model for the north central

| Variable | Beta | p-value |
| --- | --- | --- |
| Intercept | 482.240 | <0.0001 |
| Surface albedo | Not included | -- |
| AOD | -38.557 | 0.0193 |
| Cloud cover | Not included | -- |
| Dew point | Not included | -- |
| Elevation | 0.017 | <0.0001 |
| Latitude | -0.028 | <0.0001 |
| Ozone | -0.693 | <0.0001 |
| SIS | Not included | -- |
| SO_2_ | Not included | -- |
| Year |  | 0.7173 |
| 1998 | Referent | -- |
| 2000 | -5.654 | 0.6146 |
| 2002 | 48.532 | <0.0001 |
| 2004 | 1.266 | 0.9080 |
| 2006 | -45.123 | <0.0001 |
| 2008 | 28.278 | 0.0106 |
| 2010 | -18.747 | 0.1187 |
| 2012 | 12.476 | 0.2625 |
| Year * elevation |  | <0.0001 |
| Year (1998) * elevation | Referent | -- |
| Year (2000) * elevation | 0.011 | 0.0004 |
| Year (2002) * elevation | 0.006 | 0.0770 |
| Year (2004) * elevation | 0.011 | 0.0021 |
| Year (2006) * elevation | 0.009 | 0.0049 |
| Year (2008) * elevation | 0.024 | <0.0001 |
| Year (2010) * elevation | 0.014 | 0.0011 |
| Year (2012) * elevation | 0.003 | 0.3412 |
| Year * latitude |  | 0.0364 |
| Year (1998) * latitude | Referent | -- |
| Year (2000) * latitude | -0.003 | 0.4595 |
| Year (2002) * latitude | -0.019 | <0.0001 |
| Year (2004) * latitude | -0.007 | 0.0910 |
| Year (2006) * latitude | 0.013 | 0.0027 |
| Year (2008) * latitude | -0.020 | <0.0001 |
| Year (2010) * latitude | -0.002 | 0.6880 |
| Year (2012) * latitude | -0.009 | 0.0579 |

Abbreviations: AOD, aerosol optical depth; SIS, surface incoming shortwave flux; SO_2_, sulfur dioxide.

Note: A total of n=864 observations (n=108 grids for 8 years from 1998-2012) contributed to this analysis. Refer to Table 1 for variable units.

**Table 6** Final grid-level random intercept linear mixed-effects regression model for the mid-central

| Variable | Beta | p-value |
| --- | --- | --- |
| Intercept | 178.490 | 0.0002 |
| Surface albedo | -70.110 | 0.0268 |
| AOD | -258.640 | <0.0001 |
| Cloud cover | Not included | -- |
| Dew point | Not included | -- |
| Elevation | 0.026 | <0.0001 |
| Latitude | -0.050 | <0.0001 |
| Ozone | 0.195 | 0.2219 |
| SIS | 0.397 | <0.0001 |
| SO_2_ | 179912.000 | 0.5099 |
| Year |  | <0.0001 |
| 1998 | Referent | -- |
| 2000 | -33.886 | 0.0001 |
| 2002 | -89.306 | <0.0001 |
| 2004 | -96.038 | <0.0001 |
| 2006 | -69.988 | <0.0001 |
| 2008 | -60.446 | <0.0001 |
| 2010 | -73.756 | <0.0001 |
| 2012 | -100.080 | <0.0001 |
| Year * AOD |  | <0.0001 |
| Year (1998) * AOD | Referent | -- |
| Year (2000) * AOD | 193.360 | <0.0001 |
| Year (2002) * AOD | 315.170 | <0.0001 |
| Year (2004) * AOD | 326.300 | <0.0001 |
| Year (2006) * AOD | 198.820 | <0.0001 |
| Year (2008) * AOD | 213.640 | <0.0001 |
| Year (2010) * AOD | 304.870 | <0.0001 |
| Year (2012) * AOD | 282.990 | <0.0001 |
| Year * elevation |  | 0.0014 |
| Year (1998) * elevation | Referent | -- |
| Year (2000) * elevation | 0.007 | 0.0003 |
| Year (2002) * elevation | 0.009 | <0.0001 |
| Year (2004) * elevation | 0.016 | <0.0001 |
| Year (2006) * elevation | -0.001 | 0.4445 |
| Year (2008) * elevation | -0.002 | 0.3575 |
| Year (2010) * elevation | 0.000 | 0.9057 |
| Year (2012) * elevation | 0.003 | 0.1431 |
| Year * latitude |  | <0.0001 |
| Year (1998) * latitude | Referent | -- |
| Year (2000) * latitude | -0.001 | 0.6494 |
| Year (2002) * latitude | 0.017 | <0.0001 |
| Year (2004) * latitude | 0.010 | 0.0010 |
| Year (2006) * latitude | 0.019 | <0.0001 |
| Year (2008) * latitude | 0.015 | <0.0001 |
| Year (2010) * latitude | 0.011 | 0.0005 |
| Year (2012) * latitude | 0.027 | <0.0001 |

Abbreviations: AOD, aerosol optical depth; SIS, surface incoming shortwave flux; SO_2_, sulfur dioxide.

Note: A total of n=1240 observations (n=155 grids for 8 years from 1998-2012) contributed to this analysis. Refer to Table 1 for variable units.

**Table 7** Final grid-level random intercept linear mixed-effects regression model for the south central

| Variable | Beta | p-value |
| --- | --- | --- |
| Intercept | 139.530 | 0.2505 |
| Surface albedo | -100.460 | 0.0002 |
| AOD | 35.594 | 0.4656 |
| Cloud cover | 18.322 | 0.0539 |
| Dew point | Not included | -- |
| Elevation | 0.016 | <0.0001 |
| Latitude | -0.043 | <0.0001 |
| Ozone | 0.145 | 0.7257 |
| SIS | 0.442 | <0.0001 |
| SO_2_ | -825857.000 | 0.0048 |
| Year |  | 0.0534 |
| 1998 | Referent | -- |
| 2000 | -3.948 | 0.6462 |
| 2002 | -37.915 | <0.0001 |
| 2004 | -3.756 | 0.7497 |
| 2006 | -19.173 | 0.0204 |
| 2008 | -33.091 | 0.0069 |
| 2010 | 0.702 | 0.9562 |
| 2012 | 6.377 | 0.5573 |
| Year * AOD |  | 0.5518 |
| Year (1998) * AOD | Referent | -- |
| Year (2000) * AOD | 2.464 | 0.9662 |
| Year (2002) * AOD | 117.730 | 0.0325 |
| Year (2004) * AOD | -3.668 | 0.9542 |
| Year (2006) * AOD | -33.804 | 0.5211 |
| Year (2008) * AOD | 11.869 | 0.8588 |
| Year (2010) * AOD | -183.810 | 0.0087 |
| Year (2012) * AOD | -99.351 | 0.0852 |
| Year * elevation |  | 0.1363 |
| Year (1998) * elevation | Referent | -- |
| Year (2000) * elevation | 0.006 | 0.1606 |
| Year (2002) * elevation | 0.003 | 0.3548 |
| Year (2004) * elevation | 0.001 | 0.8604 |
| Year (2006) * elevation | -0.006 | 0.1053 |
| Year (2008) * elevation | -0.004 | 0.3390 |
| Year (2010) * elevation | -0.012 | 0.0205 |
| Year (2012) * elevation | -0.020 | <0.0001 |
| Year * latitude |  | 0.9992 |
| Year (1998) * latitude | Referent | -- |
| Year (2000) * latitude | -0.011 | 0.0712 |
| Year (2002) * latitude | -0.001 | 0.8101 |
| Year (2004) * latitude | -0.017 | 0.0073 |
| Year (2006) * latitude | 0.002 | 0.7845 |
| Year (2008) * latitude | 0.013 | 0.0206 |
| Year (2010) * latitude | 0.014 | 0.0197 |
| Year (2012) * latitude | 0.001 | 0.8416 |

Abbreviations: AOD, aerosol optical depth; SIS, surface incoming shortwave flux; SO_2_, sulfur dioxide.

Note: A total of n=712 observations (n=89 grids for 8 years from 1998-2012) contributed to this analysis. Refer to Table 1 for variable units.

**Table 8** Final grid-level random intercept linear mixed-effects regression model for the northeast

| Variable | Beta | p-value |
| --- | --- | --- |
| Intercept | 303.640 | 0.0018 |
| Surface albedo | -46.294 | 0.0038 |
| AOD | -37.662 | 0.0352 |
| Cloud cover | 37.656 | 0.2678 |
| Dew point | 0.674 | 0.1582 |
| Elevation | -0.008 | 0.1149 |
| Latitude | -0.024 | 0.0013 |
| Ozone | -0.659 | 0.0027 |
| SIS | 0.401 | <0.0001 |
| SO_2_ | Not included | -- |
| Year |  | 0.2661 |
| 1998 | Referent | -- |
| 2000 | 98.694 | 0.0168 |
| 2002 | 55.437 | 0.2301 |
| 2004 | -111.520 | 0.0223 |
| 2006 | -60.586 | 0.1690 |
| 2008 | -134.100 | 0.0009 |
| 2010 | -88.874 | 0.0381 |
| 2012 | -10.757 | 0.8405 |
| Year * cloud cover |  | 0.7529 |
| Year (1998) * cloud cover | Referent | -- |
| Year (2000) * cloud cover | 3.605 | 0.9392 |
| Year (2002) * cloud cover | -52.665 | 0.2991 |
| Year (2004) * cloud cover | 56.510 | 0.1827 |
| Year (2006) * cloud cover | 16.315 | 0.6808 |
| Year (2008) * cloud cover | 51.048 | 0.2883 |
| Year (2010) * cloud cover | -18.965 | 0.6497 |
| Year (2012) * cloud cover | 21.115 | 0.5899 |
| Year * dew point |  | 0.8434 |
| Year (1998) * dew point | Referent | -- |
| Year (2000) * dew point | -1.727 | 0.0011 |
| Year (2002) * dew point | 0.280 | 0.6161 |
| Year (2004) * dew point | 0.647 | 0.2725 |
| Year (2006) * dew point | 0.047 | 0.9330 |
| Year (2008) * dew point | 1.765 | 0.0015 |
| Year (2010) * dew point | 0.252 | 0.6561 |
| Year (2012) * dew point | -0.657 | 0.3245 |
| Year * latitude |  | 0.4609 |
| Year (1998) * latitude | Referent | -- |
| Year (2000) * latitude | -0.005 | 0.5395 |
| Year (2002) * latitude | -0.016 | 0.0723 |
| Year (2004) * latitude | 0.009 | 0.2645 |
| Year (2006) * latitude | 0.015 | 0.0632 |
| Year (2008) * latitude | -0.006 | 0.5220 |
| Year (2010) * latitude | 0.024 | 0.0026 |
| Year (2012) * latitude | 0.011 | 0.1866 |

Abbreviations: AOD, aerosol optical depth; SIS, surface incoming shortwave flux; SO_2_, sulfur dioxide.

Note: A total of n=304 observations (n=38 grids for 8 years from 1998-2012) contributed to this analysis. Refer to Table 1 for variable units.

**Table 9** Final grid-level random intercept linear mixed-effects regression model for the mid-Atlantic

| Variable | Beta | p-value |
| --- | --- | --- |
| Intercept | 333.760 | <0.0001 |
| Surface albedo | Not included | -- |
| AOD | 26.140 | 0.4961 |
| Cloud cover | -51.157 | <0.0001 |
| Dew point | Not included | -- |
| Elevation | -0.024 | <0.0001 |
| Latitude | -0.045 | <0.0001 |
| Ozone | -0.099 | 0.6440 |
| SIS | Not included | -- |
| SO_2_ | Not included | -- |
| Year |  | 0.0193 |
| 1998 | Referent | -- |
| 2000 | -11.198 | 0.5941 |
| 2002 | 35.736 | 0.0203 |
| 2004 | 42.352 | 0.0057 |
| 2006 | 18.300 | 0.3028 |
| 2008 | 20.282 | 0.1827 |
| 2010 | 46.956 | 0.0844 |
| 2012 | 70.569 | 0.0013 |
| Year * AOD |  | 0.0472 |
| Year (1998) * AOD | Referent | -- |
| Year (2000) * AOD | -38.186 | 0.4503 |
| Year (2002) * AOD | -119.940 | 0.0037 |
| Year (2004) * AOD | -62.648 | 0.1368 |
| Year (2006) * AOD | -45.444 | 0.2780 |
| Year (2008) * AOD | -65.563 | 0.1483 |
| Year (2010) * AOD | -97.552 | 0.1816 |
| Year (2012) * AOD | -130.680 | 0.0264 |
| Year * elevation |  | 0.7604 |
| Year (1998) * elevation | Referent | -- |
| Year (2000) * elevation | 0.015 | 0.0058 |
| Year (2002) * elevation | -0.010 | 0.0735 |
| Year (2004) * elevation | -0.003 | 0.5919 |
| Year (2006) * elevation | -0.004 | 0.4589 |
| Year (2008) * elevation | 0.011 | 0.0460 |
| Year (2010) * elevation | 0.003 | 0.6915 |
| Year (2012) * elevation | -0.021 | 0.0002 |
| Year * latitude |  | 0.2024 |
| Year (1998) * latitude | Referent | -- |
| Year (2000) * latitude | 0.004 | 0.4896 |
| Year (2002) * latitude | 0.014 | 0.0090 |
| Year (2004) * latitude | -0.019 | 0.0024 |
| Year (2006) * latitude | -0.008 | 0.2436 |
| Year (2008) * latitude | -0.005 | 0.3978 |
| Year (2010) * latitude | -0.015 | 0.0409 |
| Year (2012) * latitude | -0.019 | 0.0046 |

Abbreviations: AOD, aerosol optical depth; SIS, surface incoming shortwave flux; SO_2_, sulfur dioxide.

Note: A total of n=560 observations (n=70 grids for 8 years from 1998-2012) contributed to this analysis. Refer to Table 1 for variable units.

**Table 10** Final grid-level random intercept linear mixed-effects regression model for the southeast

| Variable | Beta | p-value |
| --- | --- | --- |
| Intercept | 450.300 | 0.0009 |
| Surface albedo | -148.160 | <0.0001 |
| AOD | 138.620 | 0.0003 |
| Cloud cover | Not included | -- |
| Dew point | -1.678 | 0.0002 |
| Elevation | -0.070 | <0.0001 |
| Latitude | -0.052 | <0.0001 |
| Ozone | -0.473 | 0.2593 |
| SIS | 0.366 | <0.0001 |
| SO_2_ | Not included | -- |
| Year |  | 0.0110 |
| 1998 | Referent | -- |
| 2000 | 23.675 | 0.0125 |
| 2002 | 28.390 | 0.0034 |
| 2004 | 6.785 | 0.4854 |
| 2006 | 11.753 | 0.1796 |
| 2008 | 26.378 | 0.0121 |
| 2010 | 24.390 | 0.0349 |
| 2012 | 25.771 | 0.0224 |
| Year * AOD |  | <0.0001 |
| Year (1998) * AOD | Referent | -- |
| Year (2000) * AOD | -138.320 | 0.0020 |
| Year (2002) * AOD | -126.380 | 0.0193 |
| Year (2004) * AOD | -116.800 | 0.0157 |
| Year (2006) * AOD | -195.300 | <0.0001 |
| Year (2008) * AOD | -191.790 | 0.0002 |
| Year (2010) * AOD | -211.430 | 0.0003 |
| Year (2012) * AOD | -283.260 | <0.0001 |
| Year * latitude |  | <0.0001 |
| Year (1998) * latitude | Referent | -- |
| Year (2000) * latitude | 0.009 | 0.0922 |
| Year (2002) * latitude | 0.012 | 0.1746 |
| Year (2004) * latitude | 0.021 | 0.0007 |
| Year (2006) * latitude | 0.022 | 0.0002 |
| Year (2008) * latitude | 0.010 | 0.0871 |
| Year (2010) * latitude | 0.022 | <0.0001 |
| Year (2012) * latitude | 0.038 | <0.0001 |

Abbreviations: AOD, aerosol optical depth; SIS, surface incoming shortwave flux; SO_2_, sulfur dioxide.

Note: A total of n=688 observations (n=86 grids for 8 years from 1998-2012) contributed to this analysis. Refer to Table 1 for variable units.

**Table 11** Goodness-of-fit for final regional random intercept linear mixed-effects regression models

| Region | R^2^ |
| --- | --- |
| Northwest | 0.85 |
| Pacific mid-West | 0.53 |
| Southwest | 0.62 |
| North central | 0.85 |
| Mid-central | 0.90 |
| South central | 0.82 |
| Northeast | 0.88 |
| Mid-Atlantic | 0.83 |
| Southeast | 0.70 |

**Table 12** July UV_Ery_ (mW/m^2^) from ATPRK, NASA grids, and UVMRP: northwest, Pacific mid-west, southwest, north central (1998-2012)

| Year | UV_Ery_ source | Region | | | | | | | | | |
| --- | --- | --- | --- | --- | --- | --- | --- | --- | --- | --- | --- |
|  |  | Northwest | Pacific mid-west | | Southwest | | North central | | | | |
|  |  | WA01 | CA01 | UT01 | CA21 | AZ01 | WI01 | MI01 | ND01 | MN01 | MT01 |
| 1998 | UVMRP | 202.27 | 242.68 | 235.84 | 260.95 | 252.40 | 207.82 | 153.62 |  | 156.90 | 194.67 |
|  | NASA | 207.39 | 241.72 | 252.67 | 272.96 | 269.29 | 188.68 | 178.79 |  | 161.13 | 191.58 |
|  | ATPRK | 200.28 | 226.79 | 217.56 | 231.68 | 252.90 | 175.79 | 177.47 |  | 163.14 | 165.82 |
| 2000 | UVMRP | 189.70 | 230.27 | 233.77 | 231.60 | 255.72 | 150.19 | 159.57 |  | 167.38 | 173.32 |
|  | NASA | 201.47 | 250.00 | 258.33 | 265.77 | 275.19 | 177.71 | 181.29 |  | 176.65 | 184.36 |
|  | ATPRK | 217.53 | 239.23 | 241.08 | 238.25 | 267.31 | 175.94 | 173.60 |  | 180.98 | 177.61 |
| 2002 | UVMRP | 193.30 | 234.53 | 227.56 | 251.45 | 248.42 | 185.40 | 188.36 |  | 160.24 |  |
|  | NASA | 210.67 | 242.76 | 264.00 | 269.68 | 262.50 | 198.23 | 197.68 |  | 179.71 |  |
|  | ATPRK | 187.64 | 226.23 | 241.52 | 237.30 | 252.63 | 188.32 | 189.56 |  | 167.22 |  |
| 2004 | UVMRP | 189.09 | 239.10 | 236.68 | 199.03 | 273.74 | 153.25 | 155.62 |  | 139.95 | 169.45 |
|  | NASA | 204.67 | 247.67 | 244.00 | 261.85 | 271.33 | 160.30 | 161.53 |  | 162.73 | 181.03 |
|  | ATPRK | 178.69 | 219.01 | 213.29 | 224.04 | 263.10 | 150.02 | 156.31 |  | 154.45 | 171.02 |
| 2006 | UVMRP | 221.20 | 236.47 | 227.37 | 241.03 | 256.74 | 171.56 | 158.50 | 176.14 | 169.09 | 186.83 |
|  | NASA | 213.36 | 246.44 | 242.88 | 254.62 | 204.37 | 177.13 | 179.57 | 201.39 | 181.08 | 194.90 |
|  | ATPRK | 205.98 | 230.22 | 218.67 | 241.79 | 190.19 | 165.90 | 170.25 | 186.17 | 174.64 | 180.33 |
| 2008 | UVMRP | 186.22 | 209.73 | 226.94 | 237.27 | 246.54 | 168.21 | 156.51 | 156.35 | 155.62 | 177.13 |
|  | NASA | 198.79 | 248.21 | 252.15 | 265.94 | 219.87 | 173.54 | 172.78 | 182.61 | 162.39 | 182.23 |
|  | ATPRK | 190.45 | 230.69 | 224.49 | 249.33 | 209.19 | 169.37 | 165.83 | 176.05 | 168.84 | 177.92 |
| 2010 | UVMRP | 196.32 | 242.03 | 228.47 | 242.00 | 234.88 | 159.52 | 156.27 | 162.63 | 155.82 | 172.55 |
|  | NASA | 191.91 | 244.89 | 236.50 | 264.43 | 226.24 | 164.08 | 174.74 | 172.52 | 158.53 | 167.88 |
|  | ATPRK | 196.07 | 227.70 | 218.41 | 242.80 | 215.32 | 150.20 | 157.11 | 163.72 | 155.16 | 172.16 |
| 2012 | UVMRP | 194.49 | 239.93 | 221.27 | 237.20 | 236.07 | 190.91 | 159.66 | 169.82 | 161.30 | 179.25 |
|  | NASA | 206.14 | 248.98 | 238.89 | 248.68 | 202.30 | 196.58 | 187.03 | 192.27 | 173.13 | 197.18 |
|  | ATPRK | 207.76 | 238.30 | 226.57 | 231.04 | 193.43 | 191.27 | 185.56 | 187.46 | 170.36 | 191.24 |

Note: Please refer to Additional File 1, Table 1 for UVMRP site locations. Blank cells indicate the station was not in operation or missing data.

Abbreviations: ATPRK, area-to-point residual kriging; NASA, National Aeronautics and Space Administration; UVMRP, UV-B Monitoring and Research Program.

**Table 13** July UV_Ery_ (mW/m^2^) from ATPRK, NASA grids, and UVMRP: mid-central and south central (1998-2012)

| Year | UV_Ery_ source | Region | | | | | | | | | | |
| --- | --- | --- | --- | --- | --- | --- | --- | --- | --- | --- | --- | --- |
|  |  | Mid-central | | | | | | | South central | | | |
|  |  | OK01 | CO41 | IL01 | IN01 | CO11 | CO01 | NE01 | TX01 | TX21 | TX41 | NM01 |
| 1998 | UVMRP |  |  | 190.90 |  |  | 210.29 | 177.58 | 247.70 |  |  | 287.63 |
|  | NASA |  |  | 198.79 |  |  | 236.67 | 192.48 | 278.57 |  |  | 274.80 |
|  | ATPRK |  |  | 169.61 |  |  | 212.48 | 160.18 | 277.86 |  |  | 284.53 |
| 2000 | UVMRP | 237.03 |  | 189.34 |  | 248.35 | 230.13 | 161.07 | 254.30 |  |  | 280.54 |
|  | NASA | 241.00 |  | 202.76 |  | 298.39 | 249.66 | 187.53 | 284.29 |  |  | 292.69 |
|  | ATPRK | 225.81 |  | 188.79 |  | 280.47 | 231.30 | 197.85 | 255.26 |  |  | 278.65 |
| 2002 | UVMRP | 209.35 |  | 185.78 | 182.73 | 267.50 | 236.83 | 191.41 | 229.90 |  |  | 252.85 |
|  | NASA | 228.62 |  | 213.82 | 218.83 | 290.00 | 256.13 | 225.52 | 247.33 |  |  | 266.67 |
|  | ATPRK | 188.89 |  | 172.57 | 172.39 | 257.40 | 215.01 | 183.49 | 256.82 |  |  | 256.23 |
| 2004 | UVMRP | 203.87 |  | 160.52 | 170.68 | 282.97 | 196.74 | 157.43 | 240.10 | 257.61 |  | 271.07 |
|  | NASA | 228.62 |  | 213.82 | 218.83 | 290.00 | 256.13 | 225.52 | 267.96 | 258.33 |  | 266.67 |
|  | ATPRK | 218.50 |  | 162.26 | 157.56 | 305.72 | 229.36 | 168.40 | 255.23 | 261.19 |  | 262.37 |
| 2006 | UVMRP | 223.27 | 243.35 | 195.60 | 166.71 | 267.44 | 207.38 | 189.21 | 263.38 | 240.14 |  | 250.32 |
|  | NASA | 234.74 | 244.01 | 199.12 | 189.34 | 296.32 | 217.37 | 214.90 | 257.72 | 236.76 |  | 239.45 |
|  | ATPRK | 212.44 | 225.81 | 170.11 | 178.04 | 289.66 | 189.00 | 176.36 | 239.93 | 243.72 |  | 233.69 |
| 2008 | UVMRP | 220.57 | 261.83 | 209.41 | 185.53 | 280.83 | 210.18 | 193.65 | 253.23 | 211.22 | 225.65 | 268.37 |
|  | NASA | 217.65 | 245.14 | 198.54 | 204.26 | 297.94 | 234.53 | 210.57 | 265.02 | 248.38 | 253.45 | 242.46 |
|  | ATPRK | 211.13 | 221.06 | 182.42 | 169.85 | 273.57 | 196.65 | 189.78 | 263.89 | 236.09 | 234.99 | 260.19 |
| 2010 | UVMRP | 229.93 | 256.37 | 195.56 | 182.55 | 266.43 | 220.81 | 175.66 |  | 229.52 | 188.00 | 266.99 |
|  | NASA | 214.70 | 249.86 | 195.09 | 187.90 | 284.57 | 240.74 | 195.35 |  | 235.01 | 212.50 | 250.91 |
|  | ATPRK | 208.49 | 222.60 | 177.25 | 151.95 | 260.26 | 199.11 | 172.59 |  | 221.77 | 207.23 | 237.28 |
| 2012 | UVMRP | 221.33 | 234.33 | 192.35 | 170.11 |  | 222.27 | 208.69 |  | 227.52 | 164.50 | 250.96 |
|  | NASA | 230.97 | 262.00 | 210.00 | 190.44 |  | 241.05 | 229.18 |  | 248.38 | 253.45 | 242.46 |
|  | ATPRK | 194.81 | 227.30 | 163.38 | 123.44 |  | 205.74 | 186.92 |  | 258.84 | 240.30 | 223.76 |

Note: Please refer to Additional File 1, Table 1 for UVMRP site locations. Blank cells indicate the station was not in operation or missing data.

Abbreviations: ATPRK, area-to-point residual kriging; NASA, National Aeronautics and Space Administration; UVMRP, UV-B Monitoring and Research Program.

**Table 14** July UV_Ery_ (mW/m^2^) from ATPRK, NASA grids, and UVMRP: northeast, mid-Atlantic, and southeast (1998-2012)

| Year | UV_Ery_ source | Region | | | | | | | | | | |
| --- | --- | --- | --- | --- | --- | --- | --- | --- | --- | --- | --- | --- |
|  |  | Northeast | | | Mid-Atlantic | | | | Southeast | | | |
|  |  | NY | VT | ME | NC01 | OH01 | MD11 | MD01 | FL01 | LA01 | MS01 | GA01 |
| 1998 | UVMRP | 154.21 | 143.66 | 137.78 |  | 177.07 |  | 177.83 | 230.81 | 213.20 |  | 171.74 |
|  | NASA | 174.65 | 155.45 | 156.07 |  | 198.79 |  | 199.48 | 256.67 | 225.63 |  | 226.41 |
|  | ATPRK | 148.00 | 133.94 | 158.45 |  | 174.08 |  | 178.91 | 231.44 | 194.53 |  | 181.08 |
| 2000 | UVMRP | 146.05 | 127.85 | 124.23 |  | 157.97 | 146.74 | 174.39 | 195.79 | 193.26 |  | 198.48 |
|  | NASA | 175.50 | 163.38 | 154.94 |  | 192.97 | 170.33 | 170.33 | 262.92 | 264.00 |  | 230.69 |
|  | ATPRK | 144.65 | 138.55 | 131.92 |  | 168.36 | 163.02 | 162.04 | 233.71 | 230.42 |  | 204.27 |
| 2002 | UVMRP | 170.52 | 142.41 | 130.77 |  |  | 161.09 | 173.37 | 208.47 | 161.54 | 191.46 | 199.85 |
|  | NASA | 194.17 | 173.52 | 144.65 |  |  | 206.39 | 206.39 | 249.05 | 246.78 | 235.16 | 229.20 |
|  | ATPRK | 148.78 | 138.32 | 141.79 |  |  | 233.72 | 233.36 | 236.29 | 240.30 | 161.70 | 196.74 |
| 2004 | UVMRP | 131.18 | 154.40 | 124.55 | 208.45 |  | 161.63 | 181.17 | 192.96 | 200.98 | 208.05 | 182.77 |
|  | NASA | 147.97 | 165.17 | 137.60 | 217.07 |  | 172.53 | 172.53 | 246.00 | 237.69 | 227.59 | 224.33 |
|  | ATPRK | 130.02 | 145.34 | 123.65 | 209.68 |  | 174.56 | 174.20 | 235.61 | 219.11 | 149.78 | 189.13 |
| 2006 | UVMRP | 170.19 | 129.88 | 145.43 | 191.71 |  | 184.34 | 185.51 | 168.61 | 174.36 | 203.66 | 193.12 |
|  | NASA | 166.67 | 159.17 | 148.46 | 203.27 |  | 179.67 | 185.22 | 227.29 | 205.84 | 217.61 | 206.16 |
|  | ATPRK | 149.60 | 130.79 | 152.99 | 195.60 |  | 177.33 | 183.10 | 214.79 | 196.30 | 200.80 | 195.86 |
| 2008 | UVMRP | 152.90 | 141.45 | 150.51 | 208.76 |  | 174.87 | 186.03 | 195.12 | 217.90 | 212.18 | 198.52 |
|  | NASA | 155.33 | 145.05 | 154.89 | 208.31 |  | 189.73 | 186.94 | 244.12 | 243.04 | 223.66 | 217.26 |
|  | ATPRK | 161.56 | 142.91 | 155.74 | 197.63 |  | 186.42 | 184.47 | 226.26 | 223.74 | 194.73 | 205.88 |
| 2010 | UVMRP | 194.41 | 154.40 | 139.76 | 189.22 |  | 177.65 | 196.37 | 180.51 | 216.15 | 236.28 | 217.30 |
|  | NASA | 173.20 | 165.75 | 166.55 | 210.59 |  | 182.89 | 190.60 | 249.60 | 244.57 | 231.40 | 222.04 |
|  | ATPRK | 134.03 | 104.23 | 145.53 | 205.53 |  | 178.44 | 183.46 | 225.96 | 221.27 | 190.27 | 193.12 |
| 2012 | UVMRP | 172.87 | 164.85 | 118.56 | 197.81 |  | 183.30 | 180.99 | 137.03 | 200.45 | 214.40 | 194.57 |
|  | NASA | 170.15 | 174.81 | 161.39 | 207.42 |  | 175.41 | 182.58 | 243.77 | 209.52 | 216.65 | 228.89 |
|  | ATPRK | 160.75 | 151.21 | 162.06 | 213.72 |  | 183.39 | 187.76 | 206.96 | 214.66 | 180.69 | 219.05 |

Note: Please refer to Additional File 1, Table 1 for UVMRP site locations. Blank cells indicate the station was not in operation or missing data.

Abbreviations: ATPRK, area-to-point residual kriging; NASA, National Aeronautics and Space Administration; UVMRP, UV-B Monitoring and Research Program.

**Table 15** Coherence property by year (1998-2012)

| Year | r_s_ | p-value |
| --- | --- | --- |
| 1998 | 0.82 | <0.0001 |
| 2000 | 0.92 | <0.0001 |
| 2002 | 0.82 | <0.0001 |
| 2004 | 0.90 | <0.0001 |
| 2006 | 0.93 | <0.0001 |
| 2008 | 0.93 | <0.0001 |
| 2010 | 0.88 | <0.0001 |
| 2012 | 0.85 | <0.0001 |

Note: A total of n=782 grids intersected the contiguous USGS U.S. states boundary layer. However, n=780 grids were included in the analysis that did not have missing elevation and/or dew point data.

**Table 16** Coherence property by region (1998-2012)

| Region | n grids^a^ | r_s_ | p-value |
| --- | --- | --- | --- |
| Northwest | 480 | 0.37 | <0.0001 |
| Pacific mid-West | 640 | 0.83 | <0.0001 |
| Southwest | 760 | 0.82 | <0.0001 |
| North central | 856 | 0.92 | <0.0001 |
| Mid-central | 1240 | 0.79 | <0.0001 |
| South central | 712 | 0.78 | <0.0001 |
| Northeast | 304 | 0.40 | <0.0001 |
| Mid-Atlantic | 560 | 0.90 | <0.0001 |
| Southeast | 688 | 0.56 | <0.0001 |

^a^This number reflects grids in all regions in all biennial years from 1998-2012.

**Table 17** Yearly NASA grid-level July UV_Ery_ descriptive statistics

|  |  | Average July UV_Ery_ (mW/m^2^) | | | | | |
| --- | --- | --- | --- | --- | --- | --- | --- |
| Year | n grids | Minimum | Mean | SD | Median | IQR | Maximum |
| 1998 | 782 | 131.27 | 225.83 | 37.42 | 225.42 | 65.23 | 308.28 |
| 1999 | 782 | 149.37 | 232.01 | 33.48 | 237.11 | 52.61 | 312.00 |
| 2000 | 782 | 139.71 | 228.48 | 40.64 | 232.50 | 71.29 | 319.17 |
| 2001 | 782 | 133.30 | 223.88 | 37.36 | 223.11 | 59.04 | 313.79 |
| 2002 | 782 | 135.13 | 230.26 | 30.42 | 230.67 | 46.58 | 316.55 |
| 2003 | 782 | 129.03 | 233.07 | 37.52 | 234.89 | 61.03 | 319.64 |
| 2004 | 782 | 137.53 | 217.35 | 39.96 | 218.33 | 64.37 | 314.00 |
| 2005 | 782 | 154.22 | 222.26 | 34.94 | 219.57 | 62.22 | 309.86 |
| 2006 | 782 | 147.40 | 215.78 | 28.61 | 217.69 | 40.58 | 311.59 |
| 2007 | 782 | 141.56 | 215.19 | 31.50 | 214.84 | 44.57 | 315.21 |
| 2008 | 782 | 136.67 | 220.35 | 32.97 | 222.62 | 51.58 | 312.87 |
| 2009 | 782 | 118.75 | 212.60 | 41.76 | 214.49 | 69.40 | 315.28 |
| 2010 | 782 | 147.73 | 215.36 | 32.39 | 219.00 | 52.31 | 307.78 |
| 2011 | 782 | 136.78 | 226.81 | 33.32 | 228.46 | 52.57 | 319.79 |
| 2012 | 782 | 153.14 | 219.92 | 28.10 | 220.55 | 42.93 | 313.30 |

Abbreviations: IQR, interquartile range; SD, standard deviation.
